# Myopalladin promotes muscle growth through modulation of the serum response factor pathway

**DOI:** 10.1002/jcsm.12486

**Published:** 2019-10-24

**Authors:** Maria Carmela Filomena, Daniel L. Yamamoto, Marco Caremani, Vinay K. Kadarla, Giuseppina Mastrototaro, Simone Serio, Anupama Vydyanath, Margherita Mutarelli, Arcamaria Garofalo, Irene Pertici, Ralph Knöll, Vincenzo Nigro, Pradeep K. Luther, Richard L. Lieber, Moriah R. Beck, Marco Linari, Marie‐Louise Bang

**Affiliations:** ^1^ Institute of Genetic and Biomedical Research (IRGB), Milan Unit National Research Council Milan Italy; ^2^ Humanitas Clinical and Research Center Rozzano Milan Italy; ^3^ Department of Biology University of Florence Sesto Fiorentino Florence Italy; ^4^ Department of Chemistry Wichita State University Wichita KS USA; ^5^ National Heart and Lung Institute Imperial College London London UK; ^6^ Telethon Institute of Genetics and Medicine (TIGEM) Pozzuoli Italy; ^7^ Department of Precision Medicine University of Campania “Luigi Vanvitelli” Naples Italy; ^8^ Integrated Cardio Metabolic Centre (ICMC), Myocardial Genetics Karolinska Institutet, University Hospital, Heart and Vascular Theme Sweden; ^9^ Research and Early Development, Cardiovascular, Renal and Metabolic Diseases (CVRM), Biopharmaceuticals R&D AstraZeneca Mölndal Sweden; ^10^ Shirley Ryan AbilityLab and Hines V.A. Medical Center Chicago Chicago IL USA; ^11^ Department of Physical Medicine and Rehabilitation Northwestern University Chicago IL USA; ^12^ Department of Orthopaedic Surgery University of California San Diego La Jolla CA USA

**Keywords:** Skeletal muscle, Sarcomere, Knockout mouse, Muscle growth, Actin dynamics, Serum response factor pathway

## Abstract

**Background:**

Myopalladin (MYPN) is a striated muscle‐specific, immunoglobulin‐containing protein located in the Z‐line and I‐band of the sarcomere as well as the nucleus. Heterozygous *MYPN* gene mutations are associated with hypertrophic, dilated, and restrictive cardiomyopathy, and homozygous loss‐of‐function truncating mutations have recently been identified in patients with cap myopathy, nemaline myopathy, and congenital myopathy with hanging big toe.

**Methods:**

Constitutive MYPN knockout (MKO) mice were generated, and the role of MYPN in skeletal muscle was studied through molecular, cellular, biochemical, structural, biomechanical, and physiological studies *in vivo* and *in vitro*.

**Results:**

MKO mice were 13% smaller compared with wild‐type controls and exhibited a 48% reduction in myofibre cross‐sectional area (CSA) and significantly increased fibre number. Similarly, reduced myotube width was observed in MKO primary myoblast cultures. Biomechanical studies showed reduced isometric force and power output in MKO mice as a result of the reduced CSA, whereas the force developed by each myosin molecular motor was unaffected. While the performance by treadmill running was similar in MKO and wild‐type mice, MKO mice showed progressively decreased exercise capability, Z‐line damage, and signs of muscle regeneration following consecutive days of downhill running. Additionally, MKO muscle exhibited progressive Z‐line widening starting from 8 months of age. RNA‐sequencing analysis revealed down‐regulation of serum response factor (SRF)‐target genes in muscles from postnatal MKO mice, important for muscle growth and differentiation. The SRF pathway is regulated by actin dynamics as binding of globular actin to the SRF‐cofactor myocardin‐related transcription factor A (MRTF‐A) prevents its translocation to the nucleus where it binds and activates SRF. MYPN was found to bind and bundle filamentous actin as well as interact with MRTF‐A. In particular, while MYPN reduced actin polymerization, it strongly inhibited actin depolymerization and consequently increased MRTF‐A‐mediated activation of SRF signalling in myogenic cells. Reduced myotube width in MKO primary myoblast cultures was rescued by transduction with constitutive active SRF, demonstrating that MYPN promotes skeletal muscle growth through activation of the SRF pathway.

**Conclusions:**

Myopalladin plays a critical role in the control of skeletal muscle growth through its effect on actin dynamics and consequently the SRF pathway. In addition, MYPN is important for the maintenance of Z‐line integrity during exercise and aging. These results suggest that muscle weakness in patients with biallelic MYPN mutations may be associated with reduced myofibre CSA and SRF signalling and that the disease phenotype may be aggravated by exercise.

## Introduction

Myopalladin (MYPN) is a 145 kDa sarcomeric protein specifically expressed in striated muscle.^1^ MYPN contains five immunoglobulin (Ig) domains and is structurally similar to the ubiquitously expressed actin‐associated protein palladin (PALLD), after which it is named.^1^, ^2^ As opposed to MYPN, PALLD is expressed in various isoforms and in particular its longest 200 kDa isoform, containing five Ig domains, is highly homologous to MYPN and predominantly expressed in striated muscle.^3^ Within the Z‐line of skeletal muscle, both MYPN and PALLD bind to α‐actinin and nebulin^1^ as well as PDZ‐LIM proteins, including Cypher/ZASP, CLP36, ALP, and RIL.^4^ In addition, MYPN is present in the nucleus and the I‐band, where it binds to the stress‐inducible protein cardiac ankyrin repeat protein (CARP/Ankrd1), which is associated with the titin N2A region in the I‐band and shuttles to the nucleus, where it functions as a transcriptional cofactor, negatively regulating muscle gene expression.^1^, ^5^

Heterozygous dominant negative *MYPN* gene mutations are associated with human hypertrophic, dilated, and restrictive cardiomyopathy (RCM).^6^, ^7^, ^8^, ^9^ Furthermore, homozygous loss‐of‐function *MYPN* truncating mutations (nonsense, frameshift, or splice‐site mutations), resulting in reduced MYPN expression, were recently identified in patients with slowly progressive cap myopathy,^10^ a relatively mild form of slowly progressive nemaline myopathy (NM) with or without intranuclear rods,^11^ and mildly progressive congenital myopathy with hanging big toe.^12^ This demonstrates the importance of MYPN in striated muscle, although its function has remained elusive.

To provide insights into the role of MYPN in skeletal muscle, we generated and studied MYPN knockout (MKO) mice. MKO mice show no signs of muscular dystrophy but have reduced myofibre cross‐sectional area (CSA), resulting in decreased isometric force and power output. Furthermore, MKO mice exhibit progressive Z‐line widening and show increased injury after downhill running. In the present study, we demonstrate that MYPN promotes skeletal muscle growth through activation of the serum response factor (SRF) signalling pathway.

## Methods

### Generation of constitutive myopalladin knockout mice

*Mypn* genomic DNA was isolated from a 129SVJ mouse genomic library (Stratagene, La Jolla, CA) and used to generate a MYPN‐targeting construct for the fusion of the endogenous *Mypn* promoter with LacZ, resulting in knockout of MYPN (Supporting Information, *Figure* S1A). The construct was created in the pBluescript II KS+ vector (Addgene), and the 5′ arm of homology consisted of a 4091 bp *BamH*I‐*Sal*I fragment upstream of the MYPN start codon in exon 2 followed by a LacZ‐neomycin cassette. The 3′ arm of homology was a 4351 *Sal*I‐*Not*I fragment downstream of the *Mypn* start codon. The targeting construct was verified by sequencing and linearized with *Not*I before electroporation into R1 ES cells at the Transgenic Core Facility at the University of California, San Diego, CA, USA. G418‐resistant ES clones were screened for homologous recombination by *Mfe*I digestion of ES cell DNA and Southern blot analysis with a 422 bp probe generated by PCR on mouse genomic DNA with *Mypn* specific primers (sense: GGAAGGCTGTAGAGCTATAAGGCATTCTAG; reverse: GCTTCAACCTTGCTATCATAGTTAAGGATG) (Supporting Information, *Figure* S1B). The wild‐type (WT) allele is represented by the band of 8041 kb, whereas the 9336 kb band represents the correctly targeted mutant allele. Cells from two independent homologous recombinant ES clones were microinjected into C57BL/6 blastocysts and transferred into pseudopregnant mice. Male chimeras resulting from the microinjections were bred with female Black Swiss mice to generate germ line transmitted heterozygous MKO mice, which were subsequently intercrossed to generate homozygous MKO mice. Offspring from intercrosses were genotyped by PCR on mouse tail DNA using WT (sense: GTTCGGTATTCTCCTCGTTTTG; reverse: CTCTGGCGGGCTGTCTTTAGG) and mutant allele‐specific primers (sense: GTTCGGTATTCTCCTCGTTTTG; reverse: AGATGAAACGCCGAGTTAACGC). Successful ablation of the *Mypn* gene was confirmed by northern blot analysis using a 1000 bp probe (sense: GGCCGCAGTACAGTTCTGAAACCCAGTCCA; reverse: TCTCTGTACCACTCGACTTTCGGAGATGGG) (Supporting Information, *Figure* S1C). Furthermore, western blot analysis using a custom‐made polyclonal antibody against MYPN^13^ (1:1000; see also Supporting Information, *Table* S2) confirmed the absence of MYPN from heart and skeletal muscle (Supporting Information, *Figure* S1D). The mice were backcrossed for 10 generations into the C57BL/6J background. All animal studies were approved by the University of California San Diego Animal Care and Use Committee and the Italian Ministry of Health. Animal procedures were performed in full compliance with the guidelines for the Guide for the Care and Use of Laboratory Animals, eighth edition (2011) published by the US National Institutes of Health and the Directive 2010/63/EU of the European Parliament on the protection of animals use for scientific purposes. Mice for experiments were sacrificed by cervical dislocation following anaesthetization by intraperitoneal (IP) injection of a mixture of ketamine (100 mg/kg) and xylazine (5 mg/kg).

### Mouse surgical procedures

For surgical procedures, mice were anaesthetized by IP injection of a mixture of ketamine (100 mg/kg) and xylazine (5 mg/kg) and the depth of anaesthesia was monitored by toe pinch. To study muscle regeneration following degeneration, 100 μL of 1.2% BaCl_2_ in 0.9% saline solution was injected into the right *tibialis anterior* (TA) muscle of 10‐week‐old male mice under general anaesthesia, while 0.9% saline solution was injected into the contralateral leg. The hindlegs were shaved before injection, allowing for better visualization of the TA. At 4, 7, 14, 21, and 28 days after injection, mice were sacrificed and TA muscles were excised and frozen in isopentane cooled with liquid nitrogen. For denervation, 10‐week‐old male mice were anaesthetized and the sciatic nerve of the left limb was cut, while the right limb served as control. Mice were sacrificed 21 days after denervation and muscles were frozen in cooled isopentane.

### Histology, immunofluorescence, and transmission electron microscopy

Transverse cryosections of 10 μm thickness of mouse hind limb muscles [TA, *extensor digitorum longus* (EDL), soleus] were subjected to haematoxylin and eosin, and Picro Sirius Red staining. Fibre‐type distribution in TA, EDL, and soleus muscle was determined by measuring myosin heavy chain (MHC) isoform levels by SDS‐PAGE as previously described.^14^ To measure CSA of myofibres of different fibre type, transverse cryosections were stained with antibodies against laminin and myosin isoform specific antibodies (Supporting Information, *Table* S2). Briefly, cross sections were incubated with primary antibodies diluted in phosphate buffered saline (PBS) for 3 days at 4°C, and subsequently with secondary antibodies for 3 h at room temperature (RT) (Supporting Information, *Table* S2). The long incubation time was necessary because the myosin isoform specific antibodies gave only a weak signal. Imaging was performed on a Leica DM IL inverted fluorescent microscope at ×10 magnification and image analyses were performed using ImageJ (National Institutes of Health). Fibre numbers were counted in mid‐belly muscle cross sections of TA, EDL, and soleus muscle.

For confocal microscopy, TA muscles were stretched, cryosectioned, and stained as previously described.^15^ The primary and secondary antibodies used are listed in Supporting Information, *Table* S2. Because the MYPN antibody^13^ was not working well for immunofluorescence staining, MYPN localization was determined by electroporation of TA muscle with PmCherry‐N1‐MYPN. A NEPA21 Electroporator (Sonidel^TM^ Limited) with a CUY560‐5‐0.5 probe was used according to the manufacturer's instructions. Confocal microscopy was performed on a Leica SP8 inverted confocal microscope with a ×60 oil immersion lens. Individual images (1024 × 1024) were converted to tiff format and merged as pseudocolour RGB images using ImageJ.

Transmission electron microscopy (TEM) was performed on EDL and soleus muscles as previously described.^13^ Z‐line density profiles were obtained using the plot profile function in ImageJ based on the analysis of >100 Z‐lines per muscle from 2–3 mice per genotype and time point and Z‐line width was measured at the base of the plots.

### Muscle mechanical analyses

#### Experiments on whole muscle

##### Force–velocity relation (Florence lab)

For mechanical studies, EDL and soleus muscles were dissected under a stereomicroscope and mounted horizontally between the lever of a motor/force transducer system (305C, Aurora Scientific Inc.) and a lever carried by a micromanipulator in a trough, containing physiological Krebs–Henseleit solution (119 mM NaCl, 4.7 mM KCl, 1.0 mM MgSO_4_, 25 mM NaHCO_3_, 1.2 mM KH_2_PO_4_, and 1.1 mM glucose), continuously saturated with carbogen (95% O_2_ and 5% CO_2_, pH 7.4) at RT (23–25°C). The muscle was straightened just above its slack length using the micromanipulator. The trough was sealed with a Perspex cover and mounted vertically on the stage with the motor/force transducer system on the top. Trains of stimuli of alternate polarity to elicit fused tetani (frequency 100–120 Hz for EDL muscle and 50–60 Hz for soleus muscle) were delivered by means of two platinum wire electrodes running parallel to the muscle, 1 cm apart. The intensity of the stimuli was increased until the isometric plateau force reached a maximum constant value, *T*_0_ (indicating that all cells in the muscle were activated). Muscle length was further finely adjusted using the micromanipulator to obtain the maximum isometric force corresponding to the plateau of the force–length relation. Isometric force was normalized to muscle wet weight (*w*) and CSA, calculated according to the relation: CSA = (*w* · cos*θ*) / (*L*_f_ · *δ*) (cm^2^), where *θ* is the pennation angle of the fibres (8.3° for EDL muscle and 8.7° for soleus muscle, table 1 in Ref. 16), *L*_f_ is the fibre length (cm), and *δ* is the muscle density (1.056 g/cm^3^)^17^. *L*_f_ was obtained by multiplying the muscle length by 0.51 in EDL muscle and 0.75 in soleus muscle (table 1 in Ref. 16). The force–velocity relation was determined by measuring the velocity of steady shortening (*V*) after a drop in force from *T*_0_ to a preset value *T* < *T*_0_ (see Supporting Information, *Figure* S3A for the protocol). The force–velocity points were fitted to the Hill hyperbolic equation^17^: (*T* + *a*) · (*V* + *b*) = (*V*_0_ + *b*) · *a*, where *a*, *b*, and *V*_0_ (unloaded shortening velocity) are the regression parameters.

##### Eccentric contraction‐induced injury (Chicago lab)

The effect of repetitive eccentric contractions of EDL muscle was tested essentially as previously described.^13^ Briefly, the fifth toe EDL muscle was dissected in Ringer's solution (137 mM NaCl, 5 mM KCl, 24 mM NaH_2_PO_4_, 2 mM CaCl_2_, 1 mM MgSO_4_, 11 mM glucose, and 1 mg/L curare) and secured in a muscle‐testing chamber, after which muscle length was adjusted by laser diffraction to a sarcomere length of ~3.0 μm. Briefly, passive tension was measured three times at 2 min intervals by imposing a 15% *L*_f_ stretch at a rate of 2 *L*_f_. Subsequently, maximum isometric force was measured three times at 5 min intervals by applying a 400 ms train of 0.3 ms pulses delivered at 100 Hz, while maintaining constant muscle length. Next, each muscle underwent a series of 10 eccentric contractions in which the muscle was first maximally activated isometrically until tension stabilized (~200 ms) and then stretched 15% *L*_f_ at a rate of 2 *L*_f_. At the end of the protocol, isometric force was again measured three times. Isometric force was normalized to the calculated CSA as mentioned earlier.

#### Experiments on skinned fibres

##### Active mechanics on single muscle fibres (Florence lab)

To investigate the effect of MYPN ablation on force per myosin motor and calcium sensitivity of the contractile apparatus, experiments were performed on glycerinated skinned fibre segments from EDL and soleus muscle essentially as described previously.^18^, ^19^ The composition of skinning, relaxing, rigour, pre‐activating, and activating solutions has been reported previously (Ref. 19 and references therein). Dissected muscles were placed in skinning solution overnight at 4°C and subsequently stored in storage solution at −20°C for up to 3–4 weeks. For mechanical testing, a muscle was transferred to a Petri dish with the bottom covered by Sylgard (Dow Corning Ltd), filled with storage solution, and kept at 4–6°C. A 2‐ to 4‐mm‐long fibre segment was cut and T‐shaped aluminum clips were mounted at its extremities for attachment to the transducer hooks. The fibre was mounted in a drop of relaxing solution between the lever arms of a loudspeaker motor and a capacitance force transducer and, to minimize the shortening of the fibre segment by extension of the damaged extremities and the development of inhomogeneity in sarcomere length across the fibre, the two ends of the fibre were fixed with glutaraldehyde in rigour solution (5% vol/vol) and glued to the clips with shellac dissolved in ethanol.^19^, ^20^ Average sarcomere length (*sl*), width (*d*), and height (*h*) of the fibre were measured in 2 to 3 points along the fibre with a ×40 dry objective (NA 0.60; Carl Zeiss MicroImaging Inc.) and a ×25 eyepiece. The *sl* of both MKO and WT fibres was set to 2.4–2.6 μm. The CSA of the fibre was determined assuming elliptical geometry (CSA = π/4·*d*·*h*), where *d* is the width and *h* is the height of the fibre.

Using a rapid solution‐exchange system, a fibre was transferred from relaxing solution to pre‐activating solution at 2°C, and after 3 min to activating solution at low temperature, where the isometric force developed is 0.1–0.2 the isometric force developed at the test temperature (12–13°C). The fibre was then transferred to activating solution at the test temperature for 6–8 s and subsequently to relaxing solution at the same temperature. This protocol prevents the development of most of the force during diffusion of Ca^2+^ and thus minimizes the development of sarcomere non‐uniformities. The solution exchange system allowed continuous recording of length changes of a selected population of sarcomeres (500–1200) by a striation follower (Ref. 19 and references therein). Baseline force in the activating solution at the test temperature was determined by superimposing on the isometric contraction a fast shortening (rise time: 3 ms, amplitude: 10% of the initial bundle length *L*_0_) that induces slack in the fibre. The half‐sarcomere strain–isometric force relation (*Y*_0_–*T*_0_) was obtained by determining fibre stiffness at different [Ca^2+^] (pCa range, 6.5–4.5) by imposing step changes (range −3 to +3 nm per half‐sarcomere) on an isometrically contracting fibre. Half‐sarcomere stiffness was estimated as the slope of the relation between the force attained at the end of the steps and the size of the step as measured at the level of the half‐sarcomere (*T*_1_ relation, Supporting Information, *Figure* S3B). The half‐sarcomere strain (*Y*_0_) was determined as the intercept of the linear regression to the *T*_1_ points with the abscissa.

To determine the force–pCa relation, isometric force (*T*_0_) at any [Ca^2+^] was normalized to *T*_0_ at saturating [Ca^2+^] (*T*_0,4.5_). The relation was fitted with the sigmoid Hill equation,^21^ allowing for calculation of the Hill coefficient (*n*_H_), indicating the steepness of the relation and thus the cooperativity in the Ca^2+^ activation process, and *pK* = pCa at *T*_0_ = 0.5·*T*_0,4.5_, a measure of the Ca^2+^ sensitivity of the contractile material.

##### Passive mechanics on single muscle fibres (Chicago lab)

Passive mechanical properties of single muscle fibres from TA and soleus muscle were determined as previously described.^14^ Briefly, TA and soleus muscles were dissected and placed in relaxing solution [59.4 mM imidazole, 86 mM KCH_4_O_3_S, 0.13 mM Ca (KCH_4_O_3_S)_2_, 10.8 mM Mg (KCH_4_O_3_S)_2_, 5.5 mM K_3_EDTA, 1 mM KH_2_PO_4_, 5.1 mM Na_2_ATP, and 50 μM leupeptin] for at least 60 min after which single fibre segments were dissected and kept in storage solution [170 mM K propionate, 5 mM K_3_EGTA, 5.3 mM MgCl_2_, 10 mM imidazole, 21.2 mM Na_2_ATP, 1 mM NaN_3_, 2.5 mM glutathione, 50 μM leupeptin, and 50% (vol/vol) glycerol] at −20°C for up to 3 weeks. All chemicals were obtained from Sigma‐Aldrich. For mechanical testing, fibre bundles were placed in relaxing solution and single fibre segments (2–3 mm in length) were carefully dissected and transferred to a mechanical testing chamber, filled with relaxing solution, in which they were tied to a force transducer and a motor. Sarcomere length was measured by transilluminating the fibre with a laser and projecting the diffraction pattern onto a photodiode array above the fibre. Muscle fibres were stretched to failure in 250 μm increments at 2 min intervals by the use of a micrometer attached to the motor. Sarcomere length and passive tension were recorded at the end of each 2 min interval, and the Young's modulus of the resulting stress–strain curve was calculated as the tangent at a sarcomere length of 3.0 μm. Hierarchical models were used for analysis of single fibres to allow inclusion of within muscle and between muscle variability.^22^

##### Data recording and analysis

In both labs, force and length signals were recorded with a multifunction I/O board (PCI‐6110, National Instruments, Austin, TX, USA), and a dedicated programme written in LabVIEW (National Instruments) was used for signal recording and analysis. Sample rate: 10 μs/point or 0.5 ms/point.

### Treadmill running

To test the exercise capacity of MKO mice, 3‐month‐old male mice were subjected to exercise endurance tests on a five‐lane treadmill for mice (2Biological Instruments) equipped with an electric grid carrying a mild electric current forcing the animals to run. Following 1 day of acclimatization (0% inclination for 10 min at 15 cm/s), mice were subjected to an uphill running test at 15% inclination. One week later, mice were subjected to a downhill running test at 15% declination for four consecutive days. For both uphill and downhill running tests, mice were warmed up for 10 min at 18 cm/s, whereafter the speed was increased by 3 cm/s every 2 min until exhaustion. The time and speed at which the mouse could not maintain sufficient speed to remain off the shock grid was recorded. Mice were sacrificed at various time points after the last downhill run and gastrocnemius and soleus muscles were processed for histological and TEM analyses. After the last run, some mice were injected with 100 μL 1% Evans blue dye in PBS at a 1% volume relative to body mass (200 μL per 20 g mouse), whereafter muscles were dissected out 24 h later and snap frozen in isopentane cooled with liquid nitrogen. Cryosections (10 μm) were fixed in cold acetone (−20°C) for 2 min, mounted with Prolong^TM^ Gold Antifade Mountant (Thermo Fisher Scientific), and visualized with the Olympus Fluoview FV1000 laser scanning confocal microscope (Olympus) at the excitation wavelength of 568 nm.

### Plasmid constructs

The plasmid constructs pXJ40‐FLAG‐human MRTF‐A^23^ and pGADT7‐human MRTF‐B^24^ were kindly provided by Dr. Carol Otey (University of North Carolina, Chapel Hill, NC, USA), while the 2FLAG‐mouse MRTF‐A construct was provided by Dr. Maria Vartiainen (University of Helsinki, Finland). MYPN and MRTF‐A cDNAs were isolated by PCR using available constructs or mouse/human cDNA as a template and cloned into pXJ40‐HA, pGADT7‐AD, pGBKT7‐BD, PmCherry‐N1, pTBSG, and/or pTBMalE3^25^ vectors using digestion cloning, Ligation Independent Cloning, or the In‐Fusion HD cloning kit (Clontech Laboratories) according to the manufacturer's instructions. Primer sequences are listed in Supporting Information, *Table* S1. All constructs were confirmed by sequencing.

### Culturing of cell lines

Human embryonic kidney 293 cells were maintained at subconfluence in Dulbecco's modified Eagle's medium (DMEM) containing 4.5 g/L glucose (Sigma‐Aldrich) supplemented with 10% heat inactivated fetal bovine serum (FBS) (Sigma‐Aldrich), 2 mM Ultraglutamine (Lonza), 10 U/mL penicillin, and 100 U/mL streptomycin (Lonza). Cells were grown at 37°C in a humidified atmosphere with 5% CO_2_. The myogenic C2C12 mouse cell line was maintained at subconfluence in DMEM containing 4.5 g/L glucose (Sigma‐Aldrich) supplemented with 20% heat inactivated FBS, 2 mM Ultraglutamine, 10 U/mL penicillin, and 100 U/mL streptomycin and grown at 37°C in a humidified atmosphere with 5% CO_2_. C2C12 cells were differentiated at 95% confluence by reducing serum levels to 2% horse serum (Thermo Fisher Scientific).

### Serum response factor‐luciferase reporter assays in C2C12 cells

C2C12 cells were plated on 12‐well dishes coated with 0.1% gelatin from porcine skin (Sigma‐Aldrich) and transiently transfected using Lipofectamine LTX (Thermo Fisher Scientific) according to the manufacturer's instructions, resulting in a transfection efficiency of approximately 70%. Cells were cotransfected with pxj40‐HA expression plasmids encoding HA‐tagged full‐length MYPN or PALLD isoform 4 (25, 50, and 100 ng) and/or FLAG‐tagged MRTF‐A (3 ng), as indicated, a pGL3 basic rat smooth muscle α‐actin promoter firefly luciferase reporter plasmid^26^ (250 ng), and the pRL‐TK plasmid (Promega) expressing *Renilla* luciferase under the control of the thymidine kinase promoter to normalize for transfection efficiency (5 ng). C2C12 myoblasts were induced to differentiate 24 h after transfection and were lysed after 48 h of differentiation. Firefly and *Renilla* luciferase activities were measured on a Synergy^TM^ H4 Multi‐Mode Microplate Reader (BioTek) using the Luc‐Pair Duo‐Luciferase Assay Kit 2.0 (GeneCopoeia) according to the manufacturer's instructions. Firefly luciferase activity was normalized to *Renilla* luciferase activity to account for variations in transfection efficiency. All experiments were performed in triplicate and repeated at least three times.

### Isolation of primary muscle cells

Primary myoblasts were isolated from the limbs of 3‐day‐old neonatal MKO and WT mice using a protocol modified from Ref. 27. Briefly, skinned mouse limbs were collected from neonatal pups, minced, and subjected to serial trypsinization (successive 25 min periods until all tissue was digested) in 0.05% trypsin solution B (Biological Industries, VWR) in PBS by continuous stirring at 37°C. Cells were collected by centrifugation at 1000×*g* for 5 min and resuspended in proliferation medium [DMEM with 4.5 g/L glucose (Sigma‐Aldrich), 20% heat inactivated FBS (Sigma‐Aldrich), 2 mM Ultraglutamine (Lonza), 10 mM Hepes (Lonza), and 10 U/mL penicillin and 100 U/mL streptomycin (Lonza)]. Following filtration of the homogenate in sequence through 100, 70, and 40 μm nylon mesh cell strainers, cells were preplated for 15 min on a tissue culture dish coated with 0.1% porcine skin gelatin (Sigma‐Aldrich), and subsequently for 1 h on an uncoated dish to remove fibroblasts. To further enrich the myoblast population, unwanted cells were removed using Feeder Removal Micro‐Beads (Miltenyi Biotec), and subsequently the Satellite Cell Isolation Kit (Miltenyi Biotec) using LS separation columns and a gentleMACS dissociator (Miltenyi Biotec) following the manufacturer's instructions. Cells were plated on a gelatin‐coated plate in proliferation medium supplemented with 10 ng/mL human basic fibroblast growth factor (Twin Helix) and 25 μM forskolin (Sigma‐Aldrich) to prevent spontaneous differentiation. After 24 h, cells were washed in PBS, and every 48 h, they were split by gentle detachment of myoblasts using 0.5 mM EDTA in PBS followed by half an hour preplating for removal of the few remaining fibroblasts. For experiments, the highly purified myoblasts were plated on gelatin‐coated six‐well plates (1.2 × 10^6^ cells/well) in proliferation medium containing 10 ng/mL basic fibroblast growth factor and induced to differentiate at 80% confluence by addition of differentiation medium [DMEM with 4.5 g/L glucose (Sigma‐Aldrich), 2% horse serum (Thermo Fisher Scientific), 2 mM Ultraglutamine (Lonza), 10 mM Hepes (Lonza), and 10 U/mL penicillin and 100 U/mL streptomycin (Lonza)].

### Experiments on primary muscle cells

To study proliferation rate of MKO and WT skeletal muscle primary cells, the Click‐iT^TM^ EdU Alexa Fluor 488 imaging kit (Thermo Fisher Scientific) was used. Briefly, MKO and WT skeletal muscle primary cells were plated on gelatin‐coated coverslips the day before treatment with 10 μM EdU for 2 h. Subsequently, cells were washed with PBS, fixed with 4% paraformaldehyde in PBS for 10 min, washed twice with 3% bovine serum albumin (BSA) (Bio‐Rad) in PBS, and permeabilized with 0.5% Triton X‐100 in PBS for 30 min at RT. After two washes with 3% BSA in PBS, cells were incubated with the Click‐iT® reaction cocktail for 30 min at RT, protected from light. Cells were then washed again with 3% BSA in PBS and incubated with Hoechst 33342 solution (5 μg/mL) for 30 min at RT. Imaging was performed on a Leica DM IL inverted fluorescent microscope at ×10 magnification and image analyses was performed using ImageJ. The number of EdU positive nuclei as a percentage of the total number of Hoechst‐stained nuclei counted in 10 fields was calculated. All experiments were performed in triplicate and repeated at least three times.

For immunofluorescence staining, cells were either untreated or infected with adenovirus expressing SRFVP16 [AdSRFVP16; 36 infectious units (ifu)]^28^, ^29^ or β‐galactosidase (AdLacZ; 10 ifu) added to the differentiation medium, after which cells were collected 40 h later. Cells were fixed with 4% paraformaldehyde for 5 min and subsequently processed for immunofluorescence staining directly in the well using a pap pen. Cells were incubated for 1 h with permeabilization/blocking solution containing 3% goat serum and 0.1% Triton X‐100 in PBS and subsequently incubated with antibodies against α‐actinin or desmin diluted in permeabilization/blocking solution overnight at 4°C (see Supporting Information, *Table* S2 for details). Following washing in 0.1% Triton X‐100 in PBS, sections were incubated with secondary antibodies for 1 h at RT. After washing, slides were incubated for 5 min with 300 nM 4′,6‐diamidino‐2‐phenylindole (DAPI; Sigma‐Aldrich) and subsequently mounted with Prolong^TM^ Gold Antifade Mountant (Thermo Fisher Scientific). Light imaging was performed on an EVOS^TM^ XL Imaging System (Thermo Fisher Scientific), while fluorescent imaging was performed on an Olympus CellR time laps microscope at ×10 magnification. Composite images were generated and the fusion index was determined as the number of nuclei within α‐actinin‐stained myotubes containing three or more nuclei as a percentage of the total number of DAPI‐stained nuclei in the same field. Myotube width of arbitrarily selected myotubes was measured using ImageJ.

For determination of protein synthesis, cells differentiated for 24 h were incubated with 1 μM puromycin dihydrochloride (Merck Millipore) for 30 min and subsequently subjected to western blot analysis using an antibody against puromycin (Supporting Information, *Table* S2).^30^ For determination of protein degradation, cells were treated with 1 μM MG262 (CliniSciences) in differentiation medium for 24 h, after which cells were collected and subjected to western blot analysis using an antibody against ubiquitinated proteins (Supporting Information, *Table* S2).

An SRF reporter assay was performed by coinfection of cells with adenovirus expressing SRF‐RE reporter‐based firefly luciferase (AdSRF‐RE luc; 40 ifu) or β‐galactosidase (AdLacZ; 10 ifu) control as well as *Renilla* luciferase (AdRen‐Luc; 10 ifu) for normalization.^31^ The virus was added to the differentiation medium containing 5% horse serum, and cells were harvested 40 h later. Firefly luciferase and *Renilla* luciferase activities were determined using the Luc‐Pair Duo‐Luciferase Assay Kit 2.0 (GeneCopoeia) as described earlier. Firefly luciferase activity was normalized to *Renilla* luciferase activity to account for variations in transfection efficiency. All experiments were performed in triplicate and repeated at least three times.

### Co‐immunoprecipitation

Human embryonic kidney 293 cells were plated on 60 mm dishes and transfected with pXJ40‐HA‐MYPN FL and/or pXJ40‐FLAG‐MRTF‐A using Lipofectamine LTX (Thermo Fisher Scientific); 48 h after transfection, cells were harvested and resuspended in 200 μL of IP buffer (20 mM Tris‐HCl pH 7.5, 150 mM NaCl, 1 mM EDTA, 1 mM EGTA, 1% Triton X‐100) supplemented with protease inhibitors (1 mM phenylmethylsulfonyl fluoride and Complete Protease Inhibitor Cocktail Tablets; Roche). After 20 min incubation on ice, the lysate was centrifuged at 14 000×*g* for 10 min at 4°C and 15% of the supernatant was added with 4× SDS protein sample buffer [100 mM Tris‐HCl pH 6.8, 40% (v/v) glycerol, 312 mM sodium dodecyl sulfate (SDS), 174 mM dithiothreitol (DTT), 0.04% (w/v) bromophenol blue] and submitted to western blot analysis with FLAG‐antibody, whereas the remaining supernatant was immunoprecipitated with 10 μg HA‐antibody overnight at 4°C with agitation (see Supporting Information, *Table* S2 for antibodies); 30 μL of Protein G Dynabeads (Novex, Thermo Fisher Scientific) were used per IP. After washing in IP buffer supplemented with protease inhibitors, the beads were mixed with lysate and first incubated for 5–10 min at RT, and subsequently for 1 h at 4°C with agitation. At the end of the incubation, the mixed beads‐lysate was washed five times with IP buffer and added with 4× SDS protein sample buffer for western blot analysis using FLAG‐antibody.

### RNA extraction and quantitative real‐time PCR

Total RNA was isolated and reverse transcribed as previously described,^32^ whereafter quantitative real‐time PCR (qRT‐PCR) was performed in triplicate with custom designed oligos (Supporting Information, *Table* SI) using the GoTaq qPCR Master Mix (Promega). A ViiA^TM^ 7 Real‐Time PCR System (Applied Biosystems) was used with the following thermal cycler conditions: 95°C for 10 min, followed by 40 cycles of 95°C for 15 s, and 60°C for 1 min. *Gapdh* was used for normalization and relative expression analysis was performed using the ∆∆Ct method.

### RNA‐sequencing and analysis

DNase I‐treated RNA was analysed on a Bioanalyzer 2100 instrument (Agilent), and only RNA samples with an RNA integrity number ≥ 8 were used for the analyses. Indexed sequencing libraries were generated from 1 μg RNA using the TruSeq Stranded Total RNA Sample Prep Kit with Ribo‐Zero Gold ribosomal RNA reduction chemistry (Illumina). Electrophoresis of the libraries on a Bioanalyzer 2100 instrument showed highest peaks at 230–270 bp. Paired‐end multiplexed sequencing of libraries (four per flow cell lane) to generate reads of 100 bp was performed on a HiSeq 1000 instrument with TruSeq SBS and PE Cluster v3 Kits (Illumina); 70–100 million reads per sample were obtained. STAR v2.5^33^ was used to align each sample's paired‐end reads to the UCSC *Mus musculus* reference genome (build GRCm38/mm10). The raw read counts were normalized with TMM implemented in the Bioconductor package ‘edgeR’^34^ and differential expression analysis of read counts was performed using glmQLFit and glmQLFTest functions. For analyses of RNA‐sequencing (RNA‐Seq) data, *P* values were corrected for multiplicity according to the Benjamini–Hochberg procedure (false discovery rate).^35^ The threshold used was false discovery rate ≤ 0.1 and log2 count per million ≥ 0. Hierarchical clustering was performed using Cluster 3.0 software,^36^ including the significantly modulated genes. Clustering was performed using the *Pearson* correlation function. The heatmap images were exported using Java TreeView software.^36^ For gene ontology (GO) analysis, the ontology database within Enrichr^37^ was used to find significant enriched GO terms (*P* ≤ 0.01). The commercial Ingenuity Pathway Analysis (http://www.qiagen.com/ingenuity) software (Qiagen) was used to determine significantly affected pathways and molecular networks. Raw data files for the RNA sequencing analysis have been deposited in the NCBI Gene Expression Omnibus under accession number GEO: GSE124763.

### Serum response factor target gene analysis

Serum response factor target genes were identified using the ‘ENCODE Transcription Factor Targets’ reported in the Harmonizome database (http://amp.pharm.mssm.edu/Harmonizome/). The SRF target gene set was subsequently reduced through overlap with the significant ChIP‐Seq SRF peaks in the C2C12 cell line [downloaded from the NCBI GEO databank (GEO: GSE36024)] that fell inside promoter regions, using the intersectBed command of BEDTools suite.^38^

### SDS‐PAGE and western blot analysis

Fibre‐type distribution in TA, EDL, and soleus muscle was determined by measuring MHC isoform levels as previously described.^14^ For western blot analysis, muscle tissue was homogenized in RIPA buffer containing 50 mM Tris‐HCl pH 7.5, 150 mM NaCl, 0.5 mM DTT, 1 mM EDTA, 1% (v/v) SDS, 1% (v/v) Triton X‐100, protease inhibitors (1 mM phenylmethylsulfonyl fluoride and Complete Protease Inhibitor Cocktail Tablets; Roche), and phosphatase inhibitors (Pierce^TM^ Phosphatase Inhibitor Mini Tablets; Thermo Fisher Scientific) using a TissueLyser II (Qiagen). Cells were lysed in standard RIPA buffer containing 0.1% SDS. Protein concentration was determined using the Bio‐Rad DC^TM^ Protein Assay Kit (Bio‐Rad) according to the manufacturer's instructions. Western blot analysis was performed using the primary and secondary antibodies listed in Supporting Information, *Table* S2. The Immobilon^TM^ Western Chemiluminescent HRP Substrate (Merck Millipore) was used and chemiluminescence was detected on a Chemidoc^TM^ MP System (Bio‐Rad). Relative protein expression was determined by densitometry using ImageJ.

### Protein expression and purification

The coding sequence of the MYPN Ig3 domain was subcloned into the pTBSG vector containing an N‐terminal His_6_‐tag and a tobacco etch virus (TEV) protease cleavage site. The coding sequences of the MYPN Ig4 and Ig3–4 domains were subcloned into the pTBMalE3 vector containing an N‐terminal MBP‐tag, followed by a His‐tag and a TEV protease cleavage site.^25^ The plasmids were transformed into BL21(DE3) cells (New England Biolabs) and proteins were expressed using auto‐induction media,^39^ starting the growth at 37°C until A_600_ = 0.6–0.8 after which the temperature was reduced to 18°C for 20–24 h. Cells were harvested by centrifugation for 20 min at 2000×*g*, resuspended in lysis buffer (50 mM Tris‐Cl, 300 mM NaCl, 10 mM imidazole, pH 7.4, and Complete Protease Inhibitor Cocktail Tablets; Roche), and lysed by sonication for 20 min. After centrifugation of the lysate at 48 000×*g* for 45 min to pellet cell debris, the supernatant was loaded onto a pre‐equilibrated Proteindex™ HiBond™ Ni‐NTA column (Marvelgent Biosciences). The column was washed twice with 50 mL wash buffer (50 mM Tris‐Cl, 300 mM NaCl, 25 mM imidazole, pH 7.4), whereafter the fusion protein was eluted with 50 mL elution buffer (50 mM Tris‐Cl, 300 mM NaCl, 250 mM imidazole, pH 7.4) at RT. The N‐terminal His_6_‐tag was cleaved overnight at 18°C in elution buffer using TEV protease. MYPN Ig4 and Ig3‐4 proteins expressed from the pTBMalE3 vector were further purified by loading onto a 5 mL pre‐packed MBPTrap™ column (GE Healthcare Life Sciences), followed by cation exchange chromatography at pH 5.5 as previously described.^40^ Protein purity was confirmed by SDS‐PAGE and the purified protein was dialysed into storage buffer (20 mM Hepes, 150 mM NaCl, 2 mM DTT, pH 7.5).

### Actin co‐sedimentation assays

Actin was purified from rabbit muscle acetone powder (Pel‐Freez Biologicals) and snap frozen as previously described.^41^ Frozen stocks of G‐actin were allowed to thaw on ice and after removal of insoluble protein by centrifugation at 15 000×*g* in a Sorvall™ microcentrifuge, actin was polymerized by addition of an equal volume of 2X F‐buffer (20 mM Tris, 200 mM KCl, 4 mM MgCl_2_, 4 mM DTT, pH 8.0) and incubation for 60 min at RT. Purified MYPN domain was centrifuged at 15 000×*g* for 30 min to pellet any insoluble protein before use. For F‐actin binding assays, various concentrations (1–30 μM) of polymerized actin were incubated with 10 μM MYPN Ig3, Ig4, or Ig3–4 domains for 1 h in a final volume of 100 μL and subsequently centrifuged at 150 000×*g* for 30 min in a Beckman Airfuge® Air‐Driven ultracentrifuge. Pellets were resuspended in 100 μL 2x SDS running buffer and 35 μL of 4x loading dye was added to both supernatants and pellets. SDS‐PAGE was performed on 15% gels and gel bands were visualized by Coomassie staining. The relative amount of MYPN in supernatants and pellets was measured by densitometry using ImageJ. Actin bundling and crosslinking was monitored as before with the modification that the centrifugation was first carried out at 5000×*g* for 10 min to pellet bundled actin and subsequently at 150 000×*g* for 30 min to pellet polymerized actin, after which supernatant and pellet fractions (low speed and high speed) were separated by SDS‐PAGE as before.

### Fluorescence microscopy of actin filament bundles

Oregon Green 488 iodoacetamide (Thermo Fisher Scientific)‐labelled G‐actin was prepared as previously described.^42^ 5 μM actin was polymerized in F‐imaging buffer (5 mM Tris‐HCl, pH 8, 100 mM KCl, 2 mM MgCl_2_, 0.2 mM DTT, 0.2 mM ATP, 0.040 mg/mL catalase, and 0.2 mg/mL glucose oxidase) for 50 min, after which MYPN Ig3 domain (5–20 μM) was added and further incubated for 50 min before mounting on a microscope. All images were collected on a Leica TCS SP5 (Leica Microsystems) confocal microscope with a ×63 oil immersion lens objective.

### Pyrene fluorescence assay

A pyrene fluorescence assay was used to measure the actin polymerization rate in the presence of MYPN domains.^43^ Gel‐filtered G‐actin was labelled with N‐(1‐pyrene)iodoacetamide (Molecular Probes) as previously reported.^43^ Pyrenyl‐actin was mixed with unlabelled G‐actin to make a 10 μM, 5% pyrene‐labelled G‐actin stock. In one reaction tube, 10 μM pyrene‐labelled G‐actin was incubated with 10 μL of 10x priming solution (10 mM EGTA, 1 mM MgCl_2_) for 2 min in a total volume of 100 μL. In a separate reaction tube, 20 μL of 10x MKEI polymerization buffer (20 mM MgCl_2_, 250 mM KCl, 10 mM EGTA, 200 mM imidazole, pH 7), 4 μL of 50x G‐buffer without CaCl_2_ (500 mM Tris, pH 8.0, 100 mM DTT, 10 mM ATP), and various concentrations of MYPN (0–20 μM) were mixed in a total volume of 200 μL. The two mixtures were combined and pyrene fluorescence was immediately measured on a fluorometre with excitation at 365 nm and emission at 385 nm until a plateau in fluorescence intensity was reached. In the G‐buffer condition, no KCl was added while all other steps were identical. Raw data were normalized by subtraction of baseline fluorescence and division by the steady‐state plateau fluorescence. The overall polymerization rate of each polymerization curve was determined by plotting the slope of the linear region of the curve and converting relative fluorescence units/s into nM actin/s.

### Actin filament depolymerization

Pyrene‐labelled F‐actin (10 μM, 5% pyrene) was prepared by incubation of pyrene‐labelled G‐actin in MKEI polymerization buffer (2 mM MgCl_2_, 50 mM KCl, 1 mM EGTA, 20 mM imidazole, pH 7) for 1 h at RT in the dark.^44^, ^45^ 2 μM pyrene‐labelled F‐actin was incubated for 30 min with various concentrations of MYPN Ig3 or Ig3–4 domain (1–20 μM) in a 200 μL reaction mixture containing 1 mM DTT and 0.2 mM ATP. Subsequently, 2 μL of 1 mM Latrunculin A (Sigma‐Aldrich) prepared in DMSO was added to the mixture and actin filament disassembly was monitored by detection of pyrene fluorescence for 600 s on a fluorometer (excitation at 365 nm and emission at 388 nm). The fluorescence signal was normalized by setting the final time point intensity of F‐actin with Latrunculin A to zero and the maximum fluorescence for each data set to 1.

### Yeast two‐hybrid assays

pGBKT‐BD bait and pGADT7‐AD prey vectors (Clontech Laboratories) containing cDNA encoding the proteins of interest were cotransformed into the Y2H Gold yeast strain (Clontech Laboratories) using the Frozen‐EZ Yeast Transformation II^TM^ kit (Zymo Research) following the manufacturer's instructions. Transfected cells were spotted on selective plates of synthetically defined (SD) medium lacking the amino acids tryptophan (Trp), leucine (Leu), histidine, and adenine containing 120 ng/mL aureobasidin A and incubated at 30°C for 3–4 days to verify interaction. Successful transformation of the two plasmids was confirmed by growth on SD/‐Trp/‐Leu plates. Possible autoactivation of the bait and the prey constructs was tested by cotransformation of the bait or prey vector with empty prey or bait vector, respectively.

### Statistical analysis

Data are represented as mean ± standard error of the mean (SEM) or standard deviation (SD) as indicated. Statistical comparisons between MKO and WT were done using the unpaired Student's *t*‐test. Simultaneous effects of genotype and another experimental variable were determined using two‐way analysis of variance (ANOVA) with *post hoc* Fisher's protected least‐significant difference analysis. *P* < 0.05 was considered significant. Statistical analysis was performed using Origin (OriginLab Corporation) software (muscle mechanics) or Prism 6 software (GraphPad).

## Results

### Myopalladin knockout mice are smaller compared with wild‐type littermates and have reduced myofibre cross‐sectional area

**Figure 1:**
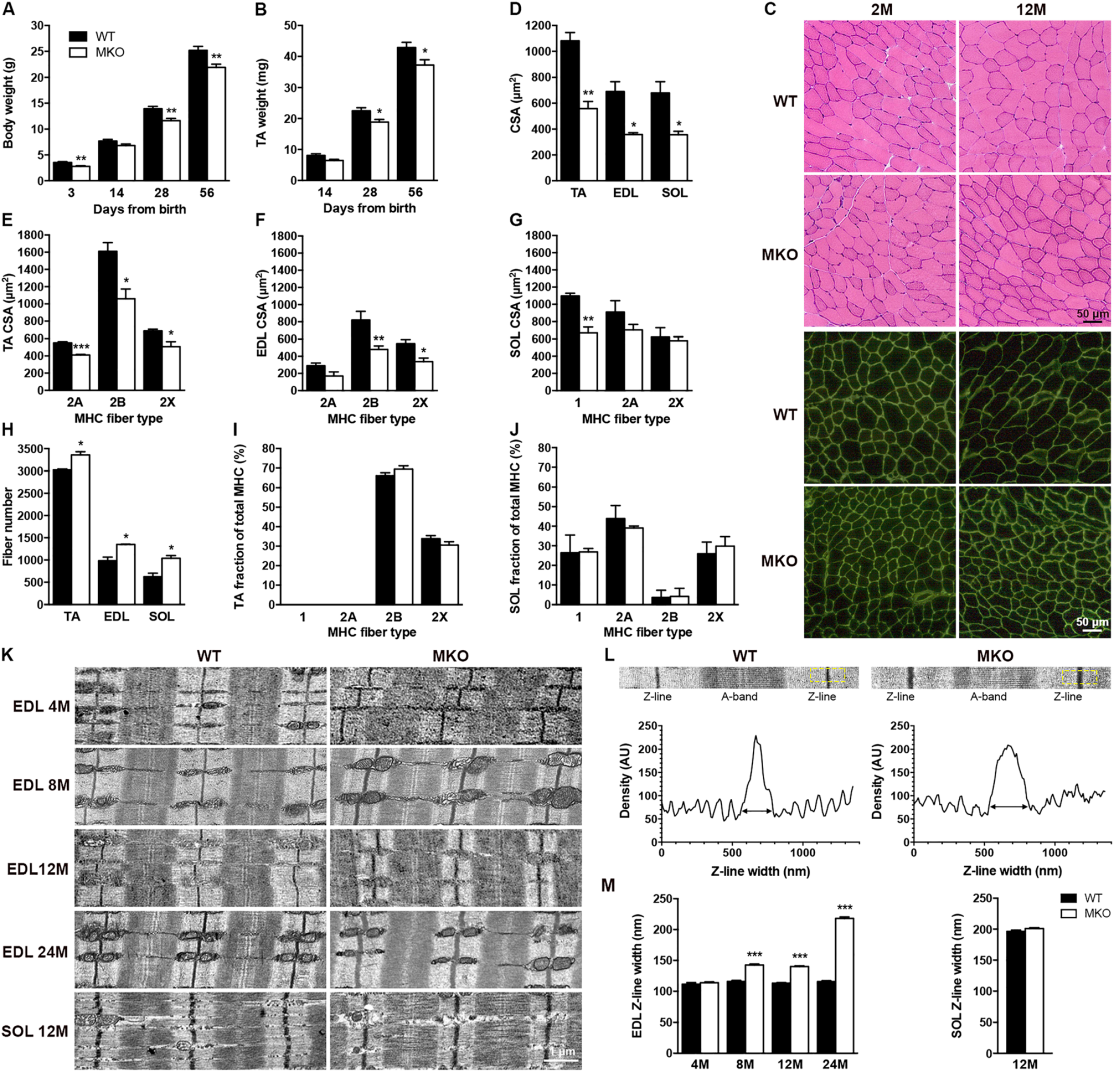
Baseline muscle characteristics of MKO and WT littermates. (A, B) Body weight (A) and TA weight (B) of MKO and WT mice at different time points after birth (*n* = 6–7 per group). (C) Haematoxylin and eosin, and laminin stainings (green) of cross‐sectioned EDL muscle from MKO and WT littermate controls of different ages. M, months. (D) Myofibre CSA of TA, EDL, and soleus (SOL) muscle from 8‐week‐old MKO and WT mice (*n* = 3 per group). (E–G) CSA of different fibre types (1, 2A, 2B, and 2X) in TA (E), EDL (F), and SOL (G) muscle (*n* = 3–5 per group). (H) Total number of fibres in mid‐belly cross sections of TA, EDL, and SOL muscle (*n* = 3 per group). (I, J) MHC fibre‐type distribution in TA (I) and SOL (J) muscle (*n* = 3 per group). See also Supporting Information, *Figure* S2A. (K) TEM of EDL and SOL muscle from MKO and WT mice of different ages showing normal structure, but progressive Z‐line widening in MKO EDL muscle from 8 months of age. Z‐line width is unaltered in SOL muscle (*n* = 2–3 per group). See Supporting Information, *Figure* S2B for low magnification pictures. (L) High magnification micrographs of single sarcomeres from 8‐month‐old MKO and WT mice showing Z‐line density profiles obtained by averaging the plots of the Z‐lines over the areas indicated by yellow boxes. Z‐line width was measured at the base of the plots as shown. (M) Z‐line width at different time points in EDL and SOL muscle. Data are represented as mean ± SEM. *P < 0.05, **P < 0.01, ***P < 0.001; Student's *t*‐test.

Constitutive MKO mice were generated by knocking in a LacZ‐Neo cassette downstream of the *Mypn* promoter, thereby disrupting the *Mypn* gene (Supporting Information, *Figure* S1). MKO mice were born at Mendelian ratios, were fertile, and had normal life spans. However, they had significantly smaller body weight compared with WT littermate control mice (22% at 3 days, 17% at 4 weeks, and 13% at 8 weeks of age) (*Figure* 1A) as well as a similar reduction in TA muscle weight (20% at 14 days, 16% at 4 weeks, and 13% at 8 weeks of age; *Figure* 1B). Histological analyses by haematoxylin and eosin staining of cryosectioned muscles showed no centralized nuclei or necrosis in MKO skeletal muscle up to 1 year of age (*Figure* 1C). However, analysis of laminin‐stained muscle sections revealed significantly reduced myofibre CSA in both fast (TA and EDL), and slow (soleus) muscles (**~**48% in all three muscles at 8 weeks of age) at all stages (*Figure* 1D and data not shown). Quantification of fibre type (1, 2A, 2B, and 2X) CSA revealed that all fibre types were affected, although there was no significant reduction in the CSA of type 2A and 2X fibres in soleus muscle (*Figure* 1E–G and Supporting Information, *Figure* S2A). The reduction in CSA of MKO muscle was associated with a significant increase in fibre number in all three muscles (11% in TA, 38% in EDL, and 66% in soleus; *Figure* 1H). SDS‐PAGE of MHC composition revealed no significant differences in fibre‐type distribution between MKO and WT mice (*Figure* 1I and 1J). Thus, reduced muscle weight in MKO mice is due to decreased myofibre CSA despite an increased myofibre number, suggesting a role of MYPN in regulating muscle growth.

### Progressive Z‐line widening in myopalladin knockout skeletal muscle

Transmission electron microscopy (TEM) of EDL muscle from MKO and WT mice at various ages revealed normal sarcomere organization in MKO muscle, but progressive Z‐line widening beginning at 8 months of age (*Figure* 1K and Supporting Information, *Figure* S2B). No abnormalities in mitochondria or other organelles were observed, but M‐line abnormalities were observed at 2 years of age. Z‐line width was increased by 23%, 24%, and 89% in MKO EDL muscle at 8, 12, and 24 months of age, respectively (*Figure* 1L and 1M), while there were no differences in I‐band width (data not shown). In contrast, no increase in Z‐line width or other ultrastructural abnormalities were observed in soleus muscle (*Figure* 1K and 1M).

### Myopalladin ablation affects specific force and power output in extensor digitorum longus muscle

**Figure 2:**
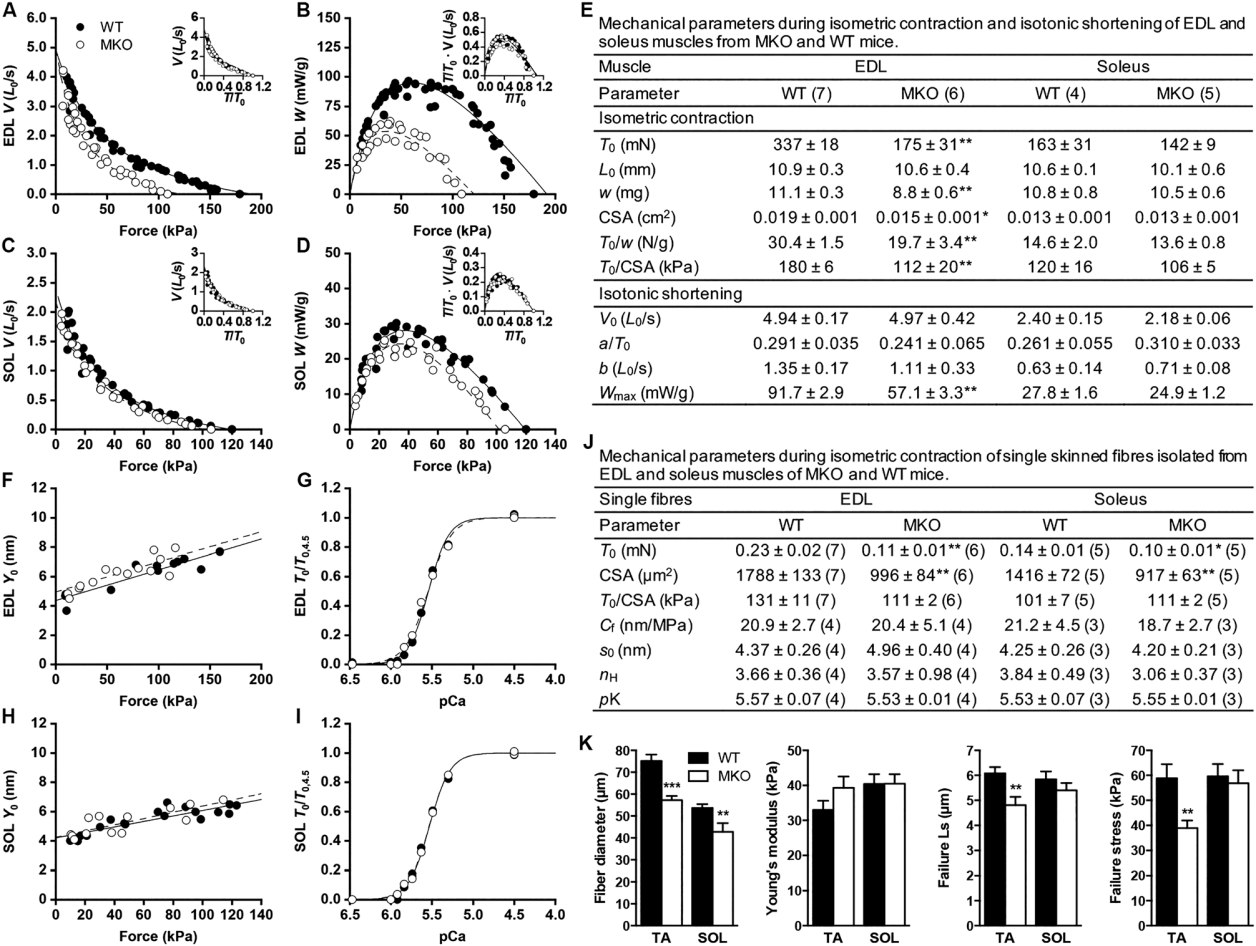
Muscle performance in 10‐week‐old MKO and WT mice. (A–E) Data from *ex* *vivo* mechanical experiments. See also Supporting Information, *Figure* S3A. (A, C) Force–velocity relations obtained from EDL (A) and soleus (SOL) (C) muscles. (B, D) Force–power relations obtained from the same data as in (A) and (C), respectively. Solid (WT) and dashed (MKO) lines are Hill's hyperbolic equations fit to the data. Inserts in (A–D), force–velocity and force–power relations with force expressed as relative to its own specific isometric force at each condition. (E) Mechanical parameters during isometric contraction and isotonic shortening of EDL and soleus muscles. Isometric contraction parameters: *T*_0_, isometric force; *L*_0_, muscle length at the plateau of the force–length relation; *w*, muscle wet weight; CSA, calculated muscle cross‐sectional area. Isotonic shortening parameters: *V*_0_, *a*/*T*_0_ and *b* are parameters of Hill's hyperbolic equation interpolated to the pooled data in (A) (EDL) and (C) (soleus); *W*_max_, maximum power obtained with a load of ~1/3·*T*_0_. (F–J) Data from single skinned muscle fibres. See also Supporting Information, *Figure* S3B. (F, H) Half‐sarcomere strain–isometric force (*Y*_0_–*T*_0_) relation obtained from fibres of EDL (F) and soleus (H) muscles. Solid (WT) and dashed (MKO) lines are linear regressions fit to the data. The parameters of the regressions [slope (C_f_) and ordinate intercept (s_0_)] are reported in (J). (G, I) Force–pCa relations obtained from fibres of EDL (G) and soleus (I) muscles. Solid (WT) and dashed (MKO) lines are Hill's sigmoidal equations fit to the data. The parameters of the Hill sigmoidal equation (steepness, *n* and Ca‐sensitivity, *p*K) are reported in (J). (J) Mechanical parameters during isometric contraction of single fibres from EDL and soleus muscles. (E, J) The numbers of samples used are shown in brackets. (K) Passive mechanical properties of single fibres from TA and soleus (SOL) muscles from MKO (*n* = 7) and WT (*n* = 8) mice. The protocol was performed in triplicate. Data are represented as mean ± SEM except for CSA in (F), which is represented as mean ± SD. *P < 0.05, **P < 0.01, ***P < 0.001; Student's *t*‐test.

To investigate the functional consequence of MYPN deletion on the mechanical properties of skeletal muscle, we measured isometric force and power output in fast (EDL) and slow (soleus) muscle from 10‐week‐old MKO and WT mice, an age at which MKO mice have normal Z‐line width and well‐preserved sarcomere structure (*Figure* 1K and 1M). In MKO EDL muscle, the isometric force (*T*_0_) was reduced by 48%, while the estimated CSA was reduced by 21%, resulting in a 38% reduction in specific isometric force (*T*_0_/CSA) (*Figure* 2E). The force–velocity relation was determined as shown in Supporting Information, *Figure* S3A. In EDL muscle, the shortening velocity at each load was significantly reduced in MKO mice compared with WT mice (*Figure* 2A), while the curvature of the force–velocity relation was not significantly altered (*Figure* 2A, inset and 2E). Consequently, the power was significantly reduced in MKO EDL muscle at any load (*Figure* 2B and 2E). The power reduction could be ascribed to the reduction in *T*_0_ in the absence of MYPN as relative power was similar in MKO and WT muscle at the same relative load (*Figure* 2B, inset). In soleus muscle, specific isometric force and power were not significantly different between genotypes (*Figure* 2C–E). The reduction in specific isometric force in MKO EDL muscle may be related to the method used to estimate the CSA, as it was indirectly measured from the muscle wet weight. To investigate this, specific isometric force was measured in fully activated single skinned fibres from EDL and soleus muscles of MKO and WT mice, where CSA was directly measured in relaxing solution. *T*_0_ was significantly reduced by 52% in EDL muscle and 29% in soleus muscle of MKO mice in respect to WT mice, while specific isometric force was comparable in both genotypes (*Figure* 2J). Thus, the reduced isometric force observed in EDL MKO muscle can be fully explained by the reduction in single fibre CSA, not accompanied by a proportional reduction in muscle wet weight.

### Myopalladin does not affect the force per myosin motor and calcium sensitivity

To investigate whether MYPN ablation affects the number of actin‐myosin motors and/or the force per myosin motor, we estimated the average strain in the myosin motors by determining the half‐sarcomere strain–force relation (*Y*_0_–*T*_0_) at different [Ca^2+^] in single skinned fibres. Both the slope and the ordinate intercept of the *Y*_0_–*T*_0_ relation were similar in MKO and WT fibres both from EDL (*Figure* 2F and 2J) and soleus muscle (*Figure* 2H and 2J), indicating that the absence of MYPN does not affect the compliance of actin and myosin filaments, nor the average strain and thus the average force per attached motor. The effect of MYPN on the Ca^2+^ sensitivity of the contractile system was tested by determining the force–pCa relation. The relations obtained from MKO and WT fibres both of EDL (*Figure* 2G) and soleus (*Figure* 2I) muscles essentially superimpose. Thus, neither cooperativity in the Ca^2+^ activation process nor Ca^2+^ myofilament sensitivity is affected by the absence of MYPN (*Figure* 2J). To determine whether MYPN affects passive mechanical properties of muscle, passive mechanical testing was performed. Young's modulus, as determined from the slope of the stress–strain curve, was not significantly different between genotypes neither in TA or soleus muscle (*Figure* 2K). However, while resting sarcomere length was similar in MKO and WT muscle, MKO fibres from TA muscle failed at a shorter sarcomere length and lower stretch, suggesting decreased structural integrity of the fibre lattice (*Figure* 2K).

### Myopalladin knockout mice are more susceptible to exercise‐induced injury

**Figure 3:**
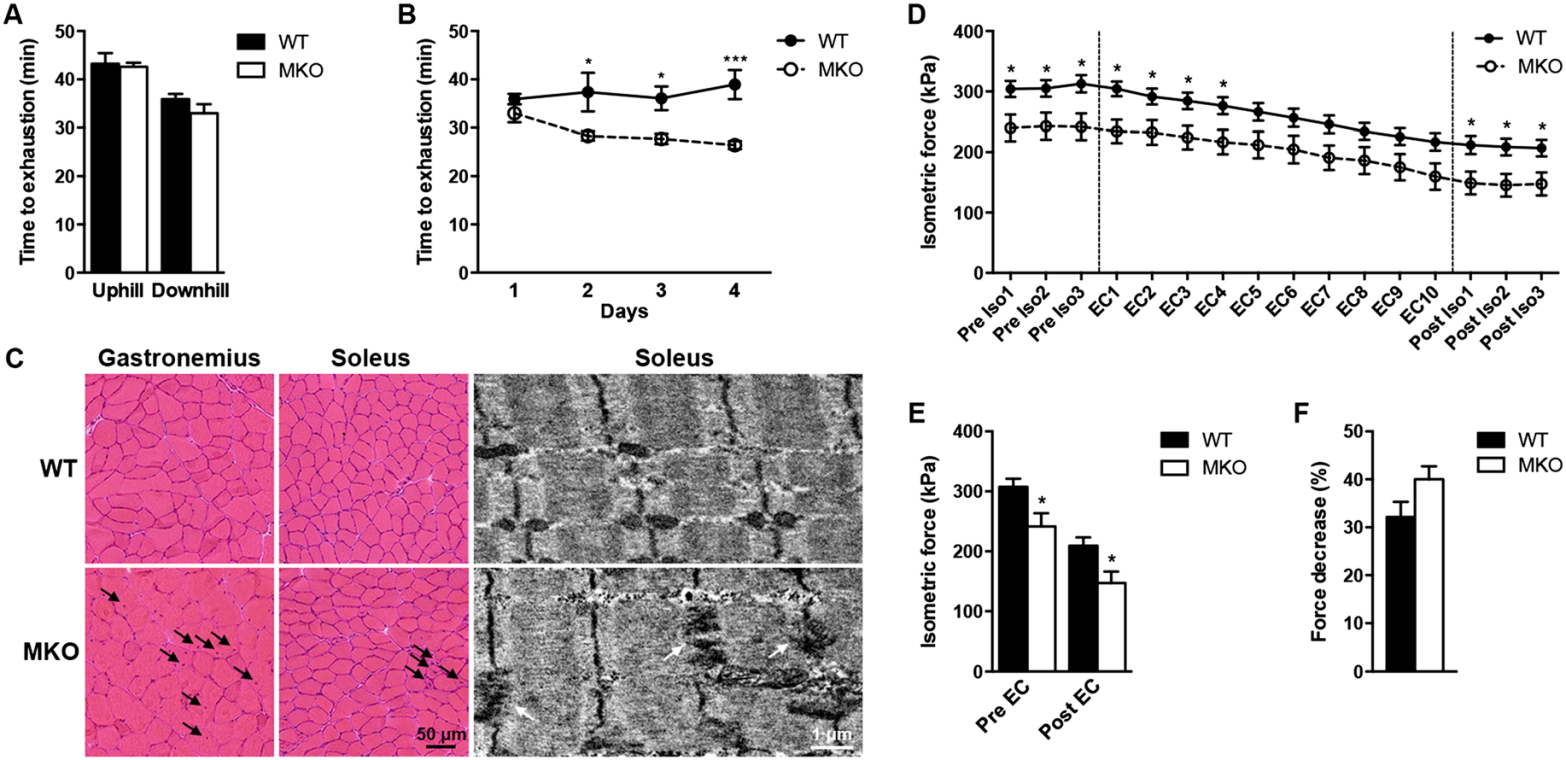
Exercise capacity of 10‐week‐old MKO and WT mice. (A) Time to exhaustion by uphill and downhill running on a treadmill (*n* = 10 per group). (B) Time to exhaustion by downhill running for four consecutive days. (C) Left, haematoxylin and eosin stainings of gastrocnemius and soleus muscles obtained 48 h after the end of the 4 day running protocol showing centralized nuclei (black arrows) in MKO mice. Right, TEM of soleus muscle obtained at the end of the 4 day running protocol, showing severe disorganization of the Z‐line (white arrows) in MKO mice. (D) Time course of isometric force before (Pre Iso1–3), during (EC1–10), and after (Post Iso1–3) cyclic eccentric exercise (EC) (demarcated by dashed lines) of EDL muscle (*n* = 6 per group). (E) Isometric force before and after EC. (F) Per cent decrease in isometric force after EC. Data are represented as mean ± SEM. *P < 0.05, ***P < 0.001; Student's *t*‐test in (A, D, E, and F) and two‐way ANOVA in (B).

To determine the exercise capacity of MKO mice, endurance tests were performed on a treadmill. No differences between genotypes were observed by uphill and downhill running on the first day of running (*Figure* 3A). However, when subjected to four consecutive days of downhill running, the performance of MKO mice was significantly reduced already after the second day of running (*Figure* 3B), suggesting that MKO mice are more susceptible to eccentric contraction‐induced muscle injury. This was supported by histological analyses of soleus and gastrocnemius muscles 48 h after the last day of treadmill running, which revealed the presence of fibres with centralized nuclei in MKO mice, a sign of regeneration (*Figure* 3C). In addition, TEM of soleus muscle showed Z‐line irregularities in MKO mice, while WT controls had normal Z‐lines and sarcomere structure (*Figure* 3C). In contrast, Evans blue staining did not show signs of membrane damage in either MKO or WT muscle (data not shown). To determine the effect of eccentric contractions *in vitro*, specific isometric force was measured before and after 10 cyclic eccentric contractions of the fifth toe EDL muscle (*Figure* 3D). As expected, it was reduced in MKO muscle compared with WT (*Figure* 3D and 3E). However, MKO and WT muscle showed similar reductions in isometric force after repetitive eccentric exercise, suggesting a similar intrinsic susceptibility to high stress.

### Myopalladin does not affect muscle regenerative capacity and is not involved in muscle wasting

**Figure 4:**
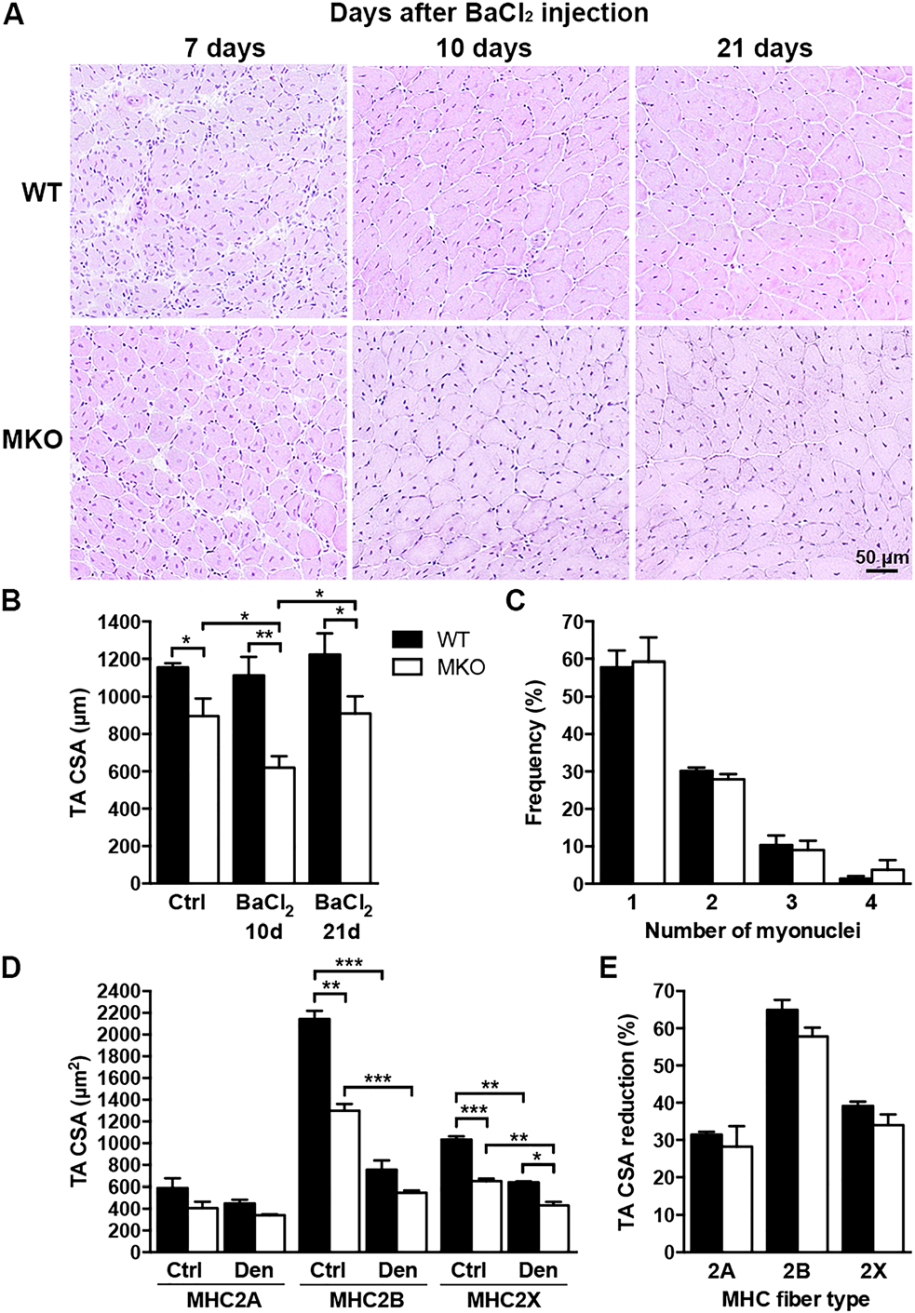
Myopalladin does not affect muscle regenerative capacity and is not involved in muscle wasting. (A) Representative hemaetoxylin and eosin stainings of TA muscle from 10‐week‐old MKO and WT mice at different time points after BaCl_2_ injection. (B) Myofibre CSA of BaCl_2_‐injected muscle *vs.* control muscle (Ctrl) 10 and 21 days (d) after injection. (C) Number of nuclei in newly formed fibres (containing centralized nuclei) 21 days after BaCl_2_ injection. (D) Myofibre CSA of different fibre types in denervated (Den) TA muscle *vs.* Ctrl muscle from 10‐week‐old MKO and WT mice. (E) Per cent reduction in CSA after denervation. (B–E) Data are represented as mean ± SEM (n = 3 per group). *P < 0.05; Student's t‐test.

To determine the ability of MKO muscle to regenerate following injury, necrosis was induced by BaCl_2_ injection into TA muscle after which the muscle regeneration process was followed histologically. MKO muscle regenerated normally (*Figure* 4A), although the CSA of regenerating fibres in MKO mice took more time to reach the CSA of the control leg (*Figure* 4B and Supporting Information, *Figure* S4). The number of nuclei in regenerating fibres was similar in BaCl_2_‐injected MKO and WT muscles, consistent with a role of MYPN in myofibre growth, but not its formation (*Figure* 4C and Supporting Information, *Figure* S4).

To determine whether MYPN may be involved in muscle wasting, we studied the effect of sciatic nerve denervation. As shown in *Figure* 4D and 4E, the reduction in TA muscle CSA after 14 days of denervation was similar in MKO and WT mice, although the initial CSA was smaller in MKO mice. Thus, MYPN does not appear to play a major role in muscle wasting.

### Reduced myotube width in primary myoblast cultures derived from myopalladin knockout mice

**Figure 5:**
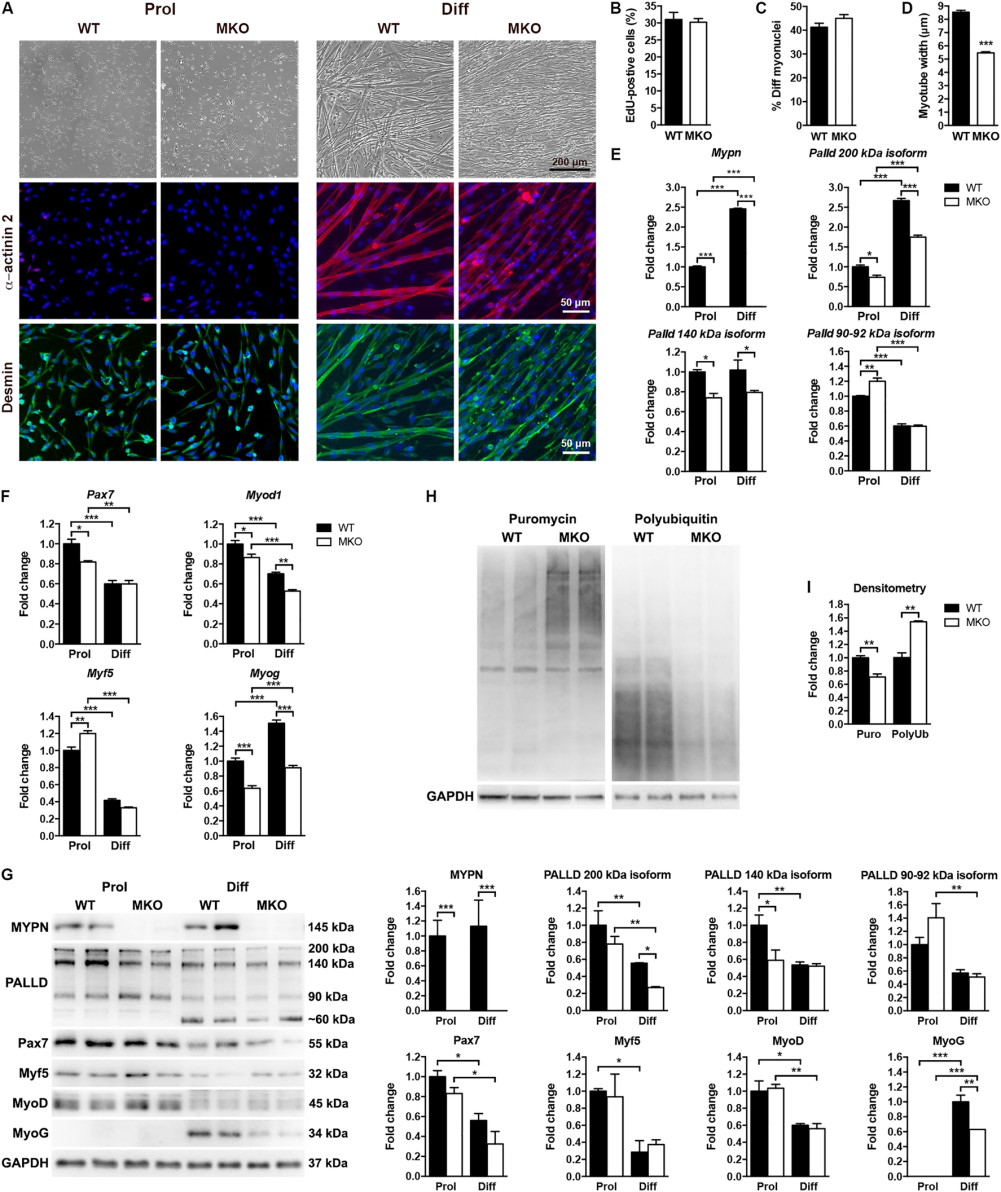
Reduced myotube width in primary MKO myoblast cultures. (A) Top, representative light microscopy images of primary myoblast cultures derived from MKO and WT mice during proliferation (Prol) and 24 h after induction of differentiation (Diff). Below, immunofluorescence staining for α‐actinin‐2 (red) and desmin (green). Nuclei are visualized by DAPI (blue). (B) Myoblast proliferation rate as measured by percentage EdU incorporation (>2200 nuclei analysed per replicate). (C) Fusion index as indicated by the percentage of myotubes containing three or more nuclei (>2500 nuclei analysed per replicate). (D) Quantification of myotube width of arbitrarily selected myotubes using ImageJ (>250 myotubes per replicate), showing reduced myotube width in MKO cultures. (E, F) qRT‐PCR analysis on proliferating (Prol) and differentiating (Diff) myoblasts from MKO and WT mice for (E) *Mypn* and *Palld* isoforms, and (F) myogenic markers. *Gapdh* was used for normalization. In (B–F), *n* = 3 per group from three independent experiments. (G) Western blot analysis for MYPN, PALLD, and myogenic markers. The blots are representatives of three replicates per group. GAPDH was used as loading control. Densitometric analyses are shown on the right. (H) Assessment of protein synthesis by western blot analysis for puromycin‐labelled proteins following puromycin‐treatment of cells (left) and protein degradation by western blot analysis for poly‐ubiquitinated proteins following MG262‐treatment (right). The blots are representatives of three replicates per group. GAPDH was used as loading control. (I) Assessment of protein synthesis and protein degradation by densitometric analysis of puromycin‐labelled (Puro) proteins and poly‐ubiquitinated (Poly‐Ub) proteins, respectively. Data are represented as mean ± SEM. *P < 0.05, **P < 0.01, ***P < 0.001; Student's *t*‐test in (B, C, and D) and two‐way ANOVA in (E, F, G, and I).

To determine whether the defective myofibre growth in MKO mice was intrinsic to muscle cells, primary myoblasts were isolated from neonatal MKO and WT mice and studied *in vitro*. While myoblast proliferation rate and fusion index were similar in MKO and WT cultures (*Figure* 5A–C), myotube width was reduced by 36% in MKO cultures 24 h after induction of differentiation (*Figure* 5A and 5D), consistent with the reduced myofibre CSA in MKO mice. In support of a role of MYPN during myoblast differentiation, a 2.5‐fold increase in mRNA levels of both MYPN and the highly homologous PALLD 200 kDa isoform was observed 24 h after induction of differentiation (*Figure* 5E). In contrast, mRNA levels of the other major PALLD isoforms were either unaltered (140 kDa isoform) or reduced (90–92 kDa isoform). At the protein level, MYPN and the 90–92 kDa PALLD isoform were expressed at similar levels in proliferating and differentiation cells, while protein expression levels of PALLD 200 and 140 kDa isoforms were reduced in differentiating cells. Interestingly, in myogenic C2C12 cells, MYPN was absent during proliferation, while both RNA and protein expression of MYPN dramatically increased during differentiation (Supporting Information, *Figure* S5A and S5B). The expression of MYPN in proliferating primary myoblasts may be due to some degree of activation of the differentiation programme. mRNA levels of both the PALLD 200 and 140 kDa isoforms were significantly reduced both in proliferating and differentiating MKO cells compared with WT cells, whereas levels of the PALLD 90–92 kDa isoform were increased in proliferating MKO myoblasts, which was also partly reflected at the protein level (*Figure* 5G). Conversely, increased mRNA levels of PALLD 200 and 140 kDa isoforms were observed in C2C12 cells overexpressing MYPN (Supporting Information, *Figure* S5C).

To further study the role of MYPN in myogenic differentiation, we determined mRNA (*Figure* 5F) and protein levels (*Figure* 5G) of myogenic markers (Pax7, MyoD, Myf5, and myogenin). *Myog* mRNA levels were strongly reduced both in proliferating and differentiating MKO cells (*Figure* 5F), resulting in a 37% reduction in myogenin expression in differentiating MKO cells (*Figure* 5G). Also, *Myod1* mRNA levels were reduced both in proliferating and differentiating MKO cells, while *Pax7* levels were decreased and *Myf5* levels increased in proliferating cells*,* although they were not significantly altered at the protein level (*Figure* 5F and 5G). Conversely, *Pax7*, *Myod1*, and *Myog* were increased in MYPN‐overexpressing C2C12 cells (Supporting Information, *Figure* S5C). Taken together, these results suggest that delayed myoblast differentiation rather than defective myoblast fusion is responsible for the smaller myofibre CSA of MKO mice.

Western blot analyses for total and phosphorylation levels of various proteins involved in muscle growth and turnover revealed no significant changes (Supporting Information, *Figure* S5D). Because muscle cell size represents a balance between protein synthesis and degradation, we measured puromycin incorporation^30^ and levels of poly‐ubiquitinated proteins after treatment with the proteasome inhibitor MG262. As shown in *Figure* 5H and 5I, a decreased amount of puromycin‐labelled peptides and an increased amount of ubiquitinylated peptides were observed in MKO cultures, indicating decreased protein synthesis and increased protein degradation in MKO cultures.

### RNA‐sequencing reveals abnormal serum response factor signalling in myopalladin knockout mice

**Figure 6:**
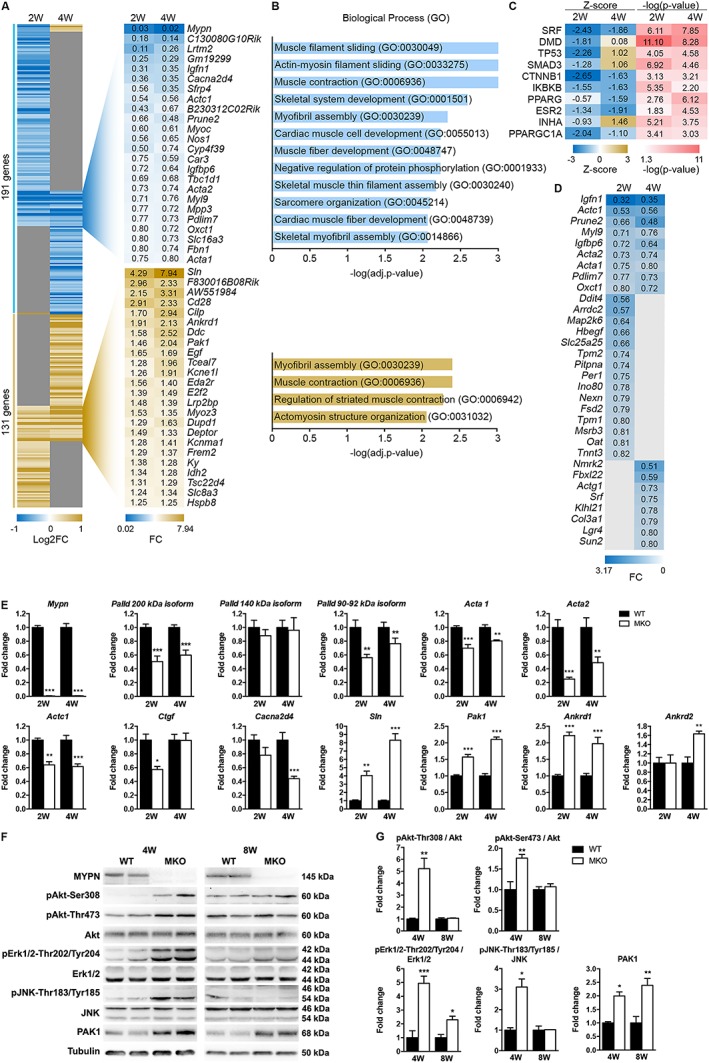
RNA‐Sequencing (RNA‐Seq) analysis reveals abnormal SRF signalling in MKO mice. (A) Heat map of unsupervised hierarchical clustering of 322 genes undergoing a change of expression (false discovery rate (FDR) ≤ 0.1; log2 count per million (logCPM) ≥0) in TA muscle from 2‐ to 4‐week‐old MKO and WT littermate control mice, respectively (*n* = 3 per group). Genes that are modulated at both time points are shown. W, weeks. See also Supporting Information, *Tables* S2 and S3. (B) Gene ontology analysis showing the significant (adj. *P* value ≤0.01) biological processes in which the commonly regulated genes are involved. (C) Ingenuity Pathway Analysis (IPA) showing the most significant upstream regulators of the modulated genes at each time point. The list is ranked by weighted scoring (‐log (adj. *P* value) *|Z‐score|). (D) Heat map showing the fold change of SRF target genes that are down‐regulated in MKO mice. (E) qRT‐PCR analysis on TA muscle from MKO and WT mice for selected transcripts confirming the RNA‐Seq results (*n* = 4–6 per group). *Gapdh* was used for normalization. (F) Western blot analysis for MYPN binding proteins and proteins involved in signalling pathways. α‐Tubulin was used as loading control. The blots are representatives of three replicates per group. See also Supporting Information, *Figure* S6B. (G) Densitometric analysis for significantly changed proteins. Data are represented as mean ± SEM. *P < 0.05, **P < 0.01, ***P < 0.001; Student's *t*‐test.

To dissect the molecular mechanisms responsible for the reduced myofibre CSA in the skeletal muscle of MKO mice, RNA‐Sequencing (RNA‐Seq) analyses were performed on TA muscle from 2‐ to 4‐week‐old MKO mice and WT littermates; 191 genes were significantly down‐regulated and 131 genes significantly up‐regulated (*Figure* 6A and Supporting Information, *Tables* S3 and S4). Hierarchical clustering of significantly modulated genes revealed a data set of 24 commonly down‐regulated genes at both time points involved in muscle contraction, muscle development, and myofibril assembly, and 24 commonly up‐regulated genes implicated in myofibril assembly and muscle contraction as well as actomyosin structure organization (*Figure* 6A and 6B). Ingenuity Pathway Analysis identified SRF as the upstream regulator of many of the down‐regulated genes (*Figure* 6C), including actin isoforms (*Acta1*, *Acta2*, and *Actc1*) and various other SRF target genes, such as *Pdlim7*, *Ctgf*, *Myl9*, *Igfbp6*, *Igfn1*, and *Fhl2* (*Figure* 6D). Among the most up‐regulated genes were the sarcomeric proteins *Sln*, *Pak1*, *Ankrd1*, and *Ankrd2*. Selected RNA‐Seq results were confirmed by qRT‐PCR (*Figure* 6E). In addition, qRT‐PCR for *Palld* isoforms revealed reduced levels of transcripts encoding PALLD 200 and 90–92 kDa isoforms in MKO muscle (*Figure* 6E). To further identify the signalling pathways affected by MYPN ablation, we performed western blot analyses for MYPN interaction partners and proteins involved in muscle metabolism (*Figure* 6F and Supporting Information, *Figure* S6). Consistent with the up‐regulation of *Pak1* in MKO mice, p21‐activated kinase 1 (PAK1) was 2.0‐fold and 2.4‐fold up‐regulated in TA muscle of 4‐ and 8‐week‐old MKO mice, respectively, while no changes in its phosphorylation level or the expression of its upstream effectors Cdc42 and Rac1/2/3 were observed (Supporting Information, *Figure* S6). Unexpectedly, increased Akt phosphorylation was observed in the muscle of 4‐week‐old MKO mice, while phosphorylation levels of the Akt downstream targets P70 S6 kinase, 4E‐BP1, and GSK3β were similar in MKO and WT muscle (Supporting information, *Figure* S6). Additionally, the mitogen‐activated protein kinases Erk1/2 and JNK were up‐regulated in MKO muscle (*Figure* 6F). RNA‐Seq showed no increase in the expression of ubiquitin ligases, such as *Atrogin1*/*Fbx032* and *Murf1*/*Trim63* in MKO mice, suggesting that the reduced CSA is not due to protein degradation through activation of the ubiquitin‐protease pathway.

### Myopalladin binds and bundles filamentous actin in vitro

**Figure 7:**
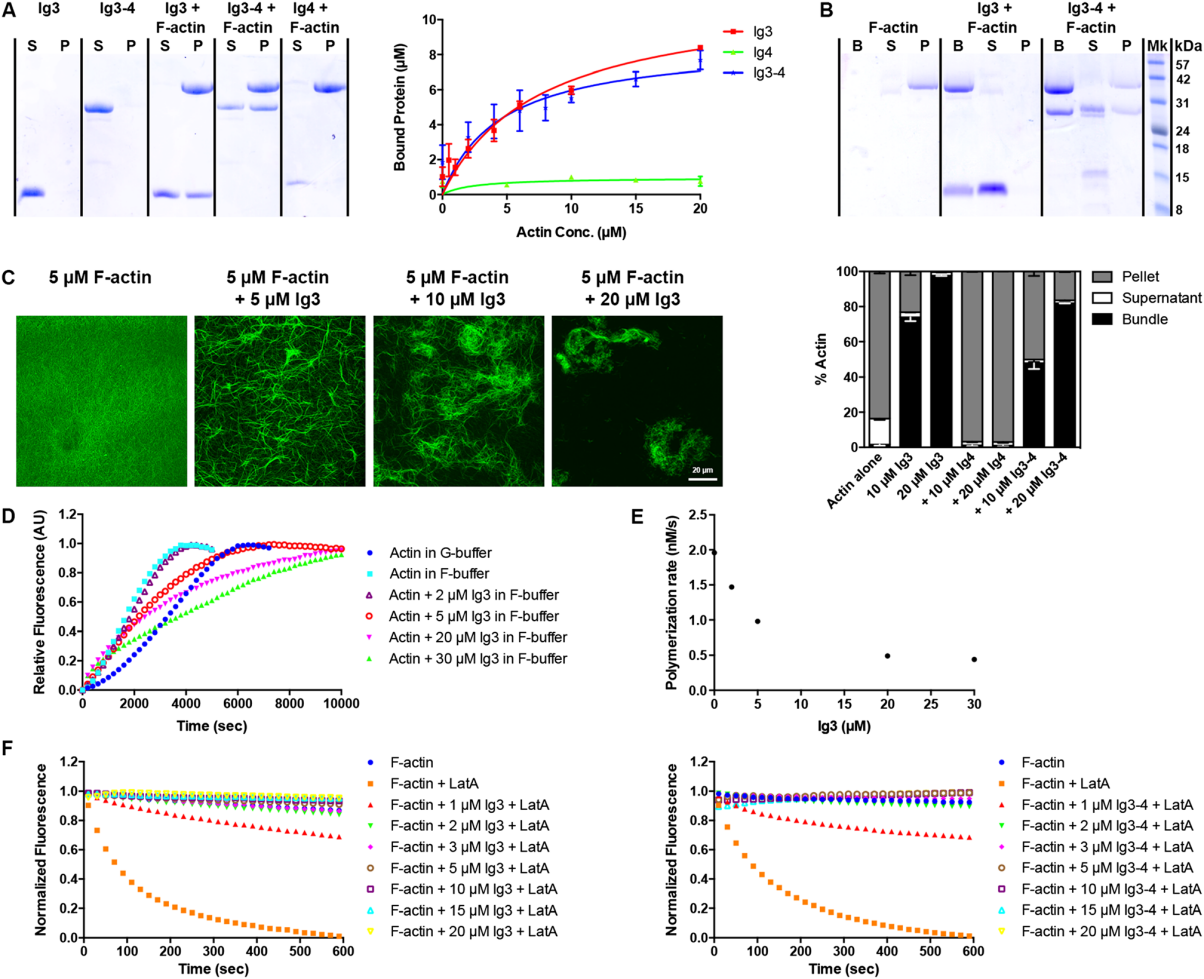
The MYPN Ig3 domain binds and bundles F‐actin. (A) Assessment of the binding of MYPN to F‐actin by ultracentrifugation of increasing concentrations of F‐actin with MYPN Ig3, Ig4, and Ig3–4 domains (10 μM), followed by SDS‐PAGE of supernatants (S) and pellets (P). The fraction of MYPN that co‐sedimented with F‐actin was determined by densitometry of the gel (*n* = 3), allowing for determination of K_d_. Data are represented as mean ± SEM. (B) Actin bundling assay showing that the MYPN Ig3 and Ig3–4 domains are capable of bundling F‐actin. F‐actin (10 μM) incubated with MYPN Ig3, Ig4, or Ig3–4 domains (10–20 μM) was subjected to low speed centrifugation to sediment bundled F‐actin. The pellet containing actin bundles (B) was collected before sedimentation of the remaining F‐actin by ultracentrifugation, whereafter SDS‐PAGE was performed. B, bundled F‐actin; S, supernatant; P, pellet. (C) Confocal images of Oregon Green‐labelled actin filaments in the presence or absence of MYPN Ig3 domain at various concentrations. (D) *In vitro* actin polymerization assay revealing that the MYPN Ig3 domain inhibits actin polymerization in a dose‐dependent manner. Pyrene‐labelled globular actin (G‐actin) (5 μM) was polymerized with MYPN Ig3 domain (2–30 μM). Polymerization was monitored by measuring the increase in fluorescence intensity over time. (E) Plot of the overall actin polymerization rate (nM/s) *vs.* MYPN Ig3 concentration. (F) *In vitro* actin depolymerization assay showing that MYPN Ig3 and Ig3–4 domains prevent F‐actin depolymerization in a dose‐dependent manner. Pyrene‐labelled F‐actin (2 μM) was incubated with MYPN Ig3 (left) or Ig3–4 domains (right) (1–20 μM) after which depolymerization was induced by addition of Latrunculin A (LatA), while measuring fluorescence intensity to monitor actin filament disassembly.

The SRF signalling pathway is regulated by changes in actin dynamics altering the intracellular ratio between globular (G‐actin) and filamentous actin (F‐actin), regulating the nuclear accumulation of the SRF coactivator myocardin‐related transcription factor A (MRTF‐A).^46^ In particular, upon actin polymerization, MRTF‐A, which is sequestered in the cytoplasm through binding to G‐actin, gets released and translocates to the nucleus where it binds and activates SRF. PALLD binds and bundles F‐actin through its Ig3 domain, homologous to the MYPN Ig3 domain, and more strongly with a fragment containing Ig3 and Ig4 (Ig3–4),^40^ thereby stabilizing actin and promoting actin polymerization and bundling.^47^ Based on the reduced SRF activity in MKO muscle and the high homology of MYPN with PALLD, we hypothesized that MYPN plays a similar role in modulating the actin cytoskeleton. To determine whether MYPN, like PALLD, can bind directly to F‐actin, we performed actin co‐sedimentation assays with MYPN Ig3, Ig4, and Ig3–4 domains. The MYPN Ig3 domain co‐sedimented with F‐actin with a dissociation constant (K_d_) of 7.6 ± 1.6 μM, while the MYPN Ig4 domain did not bind to F‐actin (*Figure* 7A). The tandem Ig3–4 domain had only slightly increased binding affinity (K_d_ MYPN Ig3–4 = 4.6 ± 2.1 μM), which is in contrast to the PALLD Ig3–4 domain, which greatly increases F‐actin binding (K_d_ = 9 ± 2 μM) compared with the PALLD Ig3 domain alone (K_d_ = 60–80 μM).^40^ Thus, the MYPN Ig3 domain exhibits a higher binding affinity towards F‐actin compared with PALLD, which is not further increased by the presence of the Ig4 domain.

To determine whether MYPN can also crosslink actin filaments, the co‐sedimentation assay was repeated with an additional low speed spin to pellet bundled F‐actin. In the absence of MYPN, F‐actin was essentially absent from the low speed pellet, whereas a significant amount of F‐actin was detected in the presence of MYPN Ig3 or Ig3–4 domains, demonstrating the ability of MYPN to crosslink F‐actin (*Figure* 7B). The presence of actin bundles was confirmed by confocal microscopy, showing dose‐dependent actin bundle formation in the presence of the MYPN Ig3 domain (*Figure* 7C). F‐actin bundling efficiency was not increased by the presence of the MYPN Ig4 domain, indicating that the MYPN Ig3 domain is sufficient for maximal crosslinking.

To monitor the effect of MYPN on actin polymerization, we measured the rate and degree of polymerization of pyrene‐labelled actin in the presence or absence of various concentrations of MYPN Ig3 domain (*Figure* 7D). In contrast to PALLD, which promotes actin polymerization,^47^ the actin polymerization rate was decreased in a dose‐dependent manner in the presence of MYPN Ig3 domain (*Figure* 7D and 7E). Unfortunately, it was not possible to reliably perform the pyrene‐actin polymerization assay with the MYPN Ig3–4 tandem domain, which appears to alter the intrinsic fluorescence of pyrene actin, presumably by binding to the actin filament, changing the environment of the pyrene. As an alternative method for measuring actin polymerization kinetics, we attempted to use dynamic light scattering, but these experiments were complicated by F‐actin bundling in the presence of MYPN, causing an increase in light scattering, thus precluding the interpretation of effects on actin polymerization.

To determine the role of MYPN in stabilizing actin filaments, we monitored the disassembly rate of pyrene‐labelled actin filaments induced by the actin monomer sequestering agent Latrunculin A in the presence of MYPN domains. Upon addition of MYPN Ig3 domain at a 1:2 molar ratio of MYPN Ig3 domain to F‐actin, the disassembly rate decreased by 80% (*Figure* 7F, left). With increasing concentrations of MYPN Ig3 domain, the rate of disassembly was further reduced and at a 1:1 ratio, the rate decreased to almost 90% compared with F‐actin alone. Similar results were observed with the MYPN Ig3–4 tandem domain (*Figure* 7F, right). These results demonstrate that binding of MYPN to actin filaments decreases the disassembly of actin monomers from filaments, thus stabilizing the filaments.

### Myopalladin binds to myocardin‐related transcriptions factors

**Figure 8:**
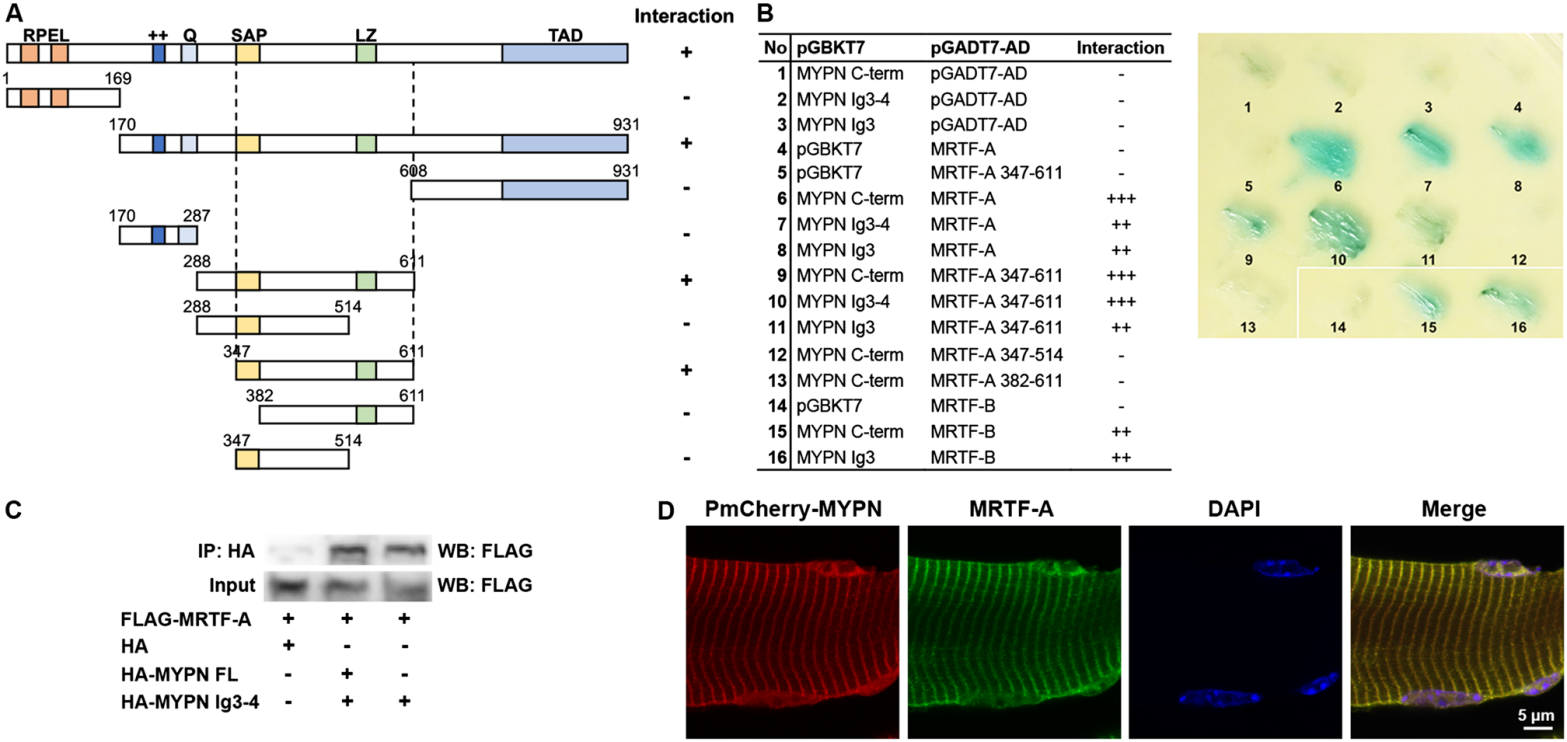
The MYPN Ig3 domain binds to myocardin‐related transcription factors (MRTFs). (A, B) Yeast‐two hybrid (Y2H) assay showing binding of the MYPN Ig3 domain to MRTF‐A residue 347–611, comprising the SAP and LZ domains. MYPN binds less strongly to MRTF‐B. (A) The domain structure of MRTF‐A is shown with the constructs that were tested for binding to MYPN in the Y2H system below. RPEL, conserved N‐terminal domain; ++, basic domain; Q, glutamine‐rich domain; SAP, SRF‐A/B‐Acinus‐PIAS domain, LZ, leucine zipper‐like domain, TAD, transactivation domain. (B) Culture plate with the different combinations of Y2H cotransformations as shown in the table on the left. (C) Co‐immunoprecipitation (Co‐IP) assay with anti‐HA antibody after transfection of HEK293 cells with HA‐tagged MYPN constructs and FLAG‐tagged MRTF‐A, confirming the binding of MYPN to MRTF‐A. The experiment was repeated three times. (D) Immunofluorescence analysis of TA muscle electroporated with PmCherry‐N1‐MYPN (red) and stained for MRTF‐A (green), showing colocalization in the sarcomere and nucleus. Nuclei are visualized by DAPI (blue).

Because PALLD is known to bind to MRTF‐A and MRTF‐B through its C‐terminal region,^48^ we used the yeast two‐hybrid (Y2H) system to test whether the corresponding region of MYPN also binds to MRTFs. As shown in *Figure* 8A and 8B, the MYPN C‐terminal region bound strongly to MRTF‐A and more weakly to MRTF‐B. Furthermore, using truncated fragments of MYPN and MRTF‐A, we found that the MYPN Ig3 domain is sufficient for binding to MRTF‐A, while the minimal binding region in MRTF‐A corresponds to residues 347–711 of MRTF‐A (Acc. NM_020831.4), including the SAP domain and the Leucine Zipper (LZ). The interaction was confirmed by co‐immunoprecipitation (*Figure* 8C) and colocalization studies (*Figure* 8D). Immunofluorescence stainings showed no differences in the localization of MRTF‐A or other MYPN‐interacting proteins in TA muscle from MKO and WT mice (Supporting Information, *Figure* S8).

### Myopalladin promotes myocardin‐related transcription factor‐induced serum response factor signalling in C2C12 cells

**Figure 9:**
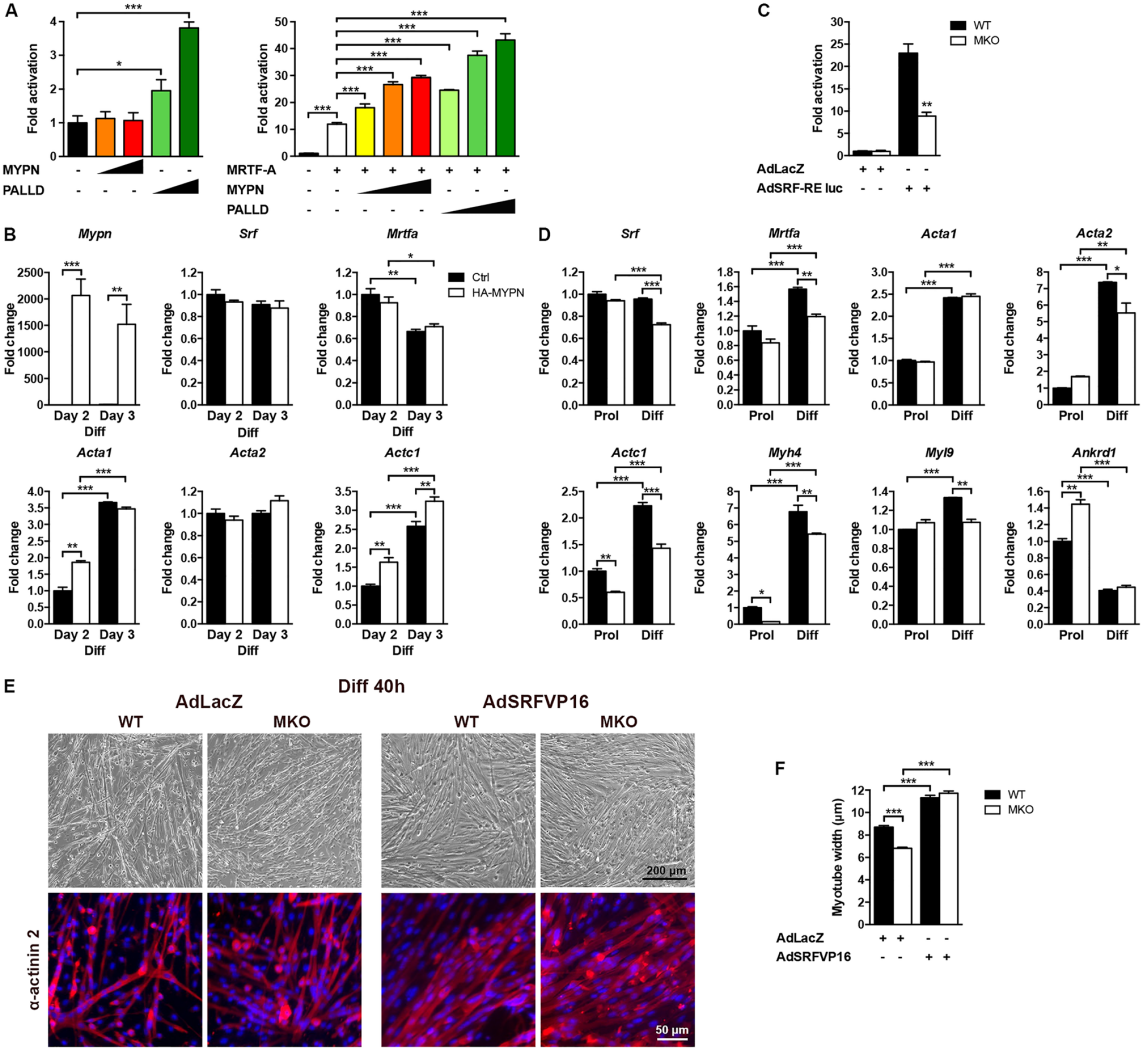
MYPN promotes SRF signalling. (A) SRF‐luciferase reporter assays in C2C12 cells, demonstrating that MYPN and PALLD increase MRTF‐A‐induced activation of SRF activity in a dose‐dependent manner. Cells were cotransfected with *Acta2* luciferase reporter and expression vectors encoding MYPN or PALLD in increasing amounts in the absence (left; 50, 100 ng of MYPN or PALLD) or presence (right; 10, 25, 50 ng of MYPN or PALLD) of MRTF‐A (3 ng). (B) qRT‐PCR on C2C12 cells 2 and 3 days after transfection with MYPN or control vector and induction of differentiation (Diff), showing up‐regulation of SRF target gene expression in MYPN‐overexpressing cells. *Gapdh* was used for normalization. (C) SRF luciferase reporter assay in primary myoblast cultures derived from MKO and WT mice performed 24 h after adenoviral infection with *Acta2* luciferase reporter and *Renilla* standardization reporter plasmids and induction of differentiation. AdLacZ was used as control. (D) qRT‐PCR on proliferating (Prol) and differentiating (Diff) myoblasts from MKO and WT mice, showing down‐regulation of SRF target gene expression in MKO cultures. *Gapdh* was used for normalization. (E) Representative light microscopy images (top) and immunofluorescence stainings (bottom) for α‐actinin‐2 (red) of primary myoblast cultures derived from MKO and WT mice 40 h after infection with Ad‐SRF‐VP16 (expressing constitutive active SRF) or Ad‐LacZ and induction of differentiation. Nuclei are visualized by DAPI (blue). See also Supporting Information, *Figure* S8. (F) Quantification of myotube width of arbitrarily selected myotubes using ImageJ 40 h after infection with Ad‐SRF‐VP16 or AdLacZ (*n* > 130 myotubes per replicate), showing rescue of reduced myotube width in MKO cultures infected with Ad‐SRF‐VP16. Three replicates per group from three independent experiments were analysed. Data are represented as mean ± SEM. *P < 0.05, **P < 0.01, ***P < 0.001; Student's *t*‐test in (C) and two‐way ANOVA in (B, D, and F).

To determine whether MYPN, like PALLD, can activate the SRF signalling pathway, we performed SRF‐luciferase reporter assays 48 h after cotransfection of myogenic C2C12 cells with increasing amounts of HA‐MYPN or HA‐PALLD in the presence and absence of FLAG‐MRTF‐A. MYPN increased MRTF‐A‐mediated activation of SRF activity in a dose‐dependent manner (*Figure* 9A), which was consistent with increased *Acta1* and *Actc1* mRNA levels, while *Mrtfa* and *Srf* levels were unaltered (*Figure* 9B). Conversely, an SRF‐luciferase assay in primary MKO and WT myoblast cultures showed reduced SRF activity in differentiating MKO cultures (*Figure* 9C). Consistently, the SRF target genes *Acta2*, *Actc1*, *Myh4*, *Myl9*, *Mrtfa*, and *Srf*, which is its own target, were down‐regulated in differentiating MKO cells, and *Actc1* and *Myh4* also during proliferation (*Figure* 9D). *CARP*/*Ankrd1*, which is also an SRF target, was up‐regulated in proliferating MKO myoblasts.

SRF is known to regulate cell growth and differentiation,^46^ and skeletal muscle specific deletion of SRF was reported to result in postnatal lethality due to a failure of muscle fibres to grow without affecting myoblast fusion.^49^ Therefore, to determine whether decreased SRF activity may be responsible for the reduced myofibre CSA in MKO muscle and consequent reduced myotube width in primary MKO cultures, we infected MKO cultures with adenovirus expressing constitutive active SRF (SRF‐VP16).^28^, ^29^ This was sufficient to rescue the MKO phenotype, resulting in similar myotube width in MKO and WT cultures, which was increased compared with the control cultures transduced with LacZ control virus (*Figure* 9E and 9F and Supporting Information, *Figure* S8).

## Discussion

In the present work, we studied the effect of MYPN ablation on skeletal muscle structure and function. MKO mice had reduced body weight and showed decreased myofibre CSA, partly compensated for by an increase in fibre number. Consistently, reduced myotube width was observed in primary skeletal muscle cultures from MKO mice compared with WT control mice. As a direct result of the reduced myofibre CSA, isometric force and power output were reduced in MKO EDL muscle, while the force developed by each myosin motor, the kinetics of actin‐myosin interaction, and the Ca^2+^‐sensitivity of the contractile apparatus were unaffected. The smaller effect of MYPN ablation on power output in soleus muscle compared with EDL muscle may be explained by (i) the reduced CSA of the fast and powerful type 2B and 2X fibres in EDL muscle (*Figure* 1F), whereas in soleus muscle, only the slow type 1 fibres were significantly reduced in size (*Figure* 1G), and (ii) the larger increase in fibre number in soleus muscle (66%) compared with EDL muscle (38%) (*Figure* 1H), possibly explained by the higher content of satellite cells and thus myogenic capacity of soleus muscle.^50^

RNA‐Seq revealed the down‐regulation of various genes involved in sarcomere assembly and organization in MKO mice, including actin isoforms and other SRF‐target genes, consistent with reduced SRF activity in MKO muscle. Among the down‐regulated SRF target genes were *Palld* isoforms, suggesting that PALLD is unlikely to compensate for MYPN loss. The effect of MYPN on the SRF pathway was confirmed by SRF luciferase assays in myogenic cells in which we showed that MYPN promotes MRTF‐A‐induced activation of SRF in a dose‐dependent manner, while MYPN ablation reduces SRF activity. Furthermore, consistent with the hypothesis that MYPN promotes skeletal muscle growth through activation of the SRF pathway, transduction of MKO myoblasts with constitutive active SRF rescued the reduced myotube width.

Because the SRF pathway is regulated by changes in actin dynamics, affecting the balance between G‐actin and F‐actin,^46^ we performed biochemical assays to determine whether MYPN, like PALLD, plays a role in organizing the actin cytoskeleton. This revealed that the MYPN Ig3 domain can bind and bundle F‐actin. However, unlike PALLD, which promotes actin polymerization,^47^ MYPN reduces the actin polymerization rate. On the other hand, it efficiently stabilizes F‐actin, even more strongly than PALLD, preventing its depolymerization. These results are consistent with the SRF luciferase assays, showing that PALLD activates SRF more strongly than MYPN, activating SRF both in the presence and absence of exogenous MRTF‐A (*Figure* 9A).

**Figure 10:**
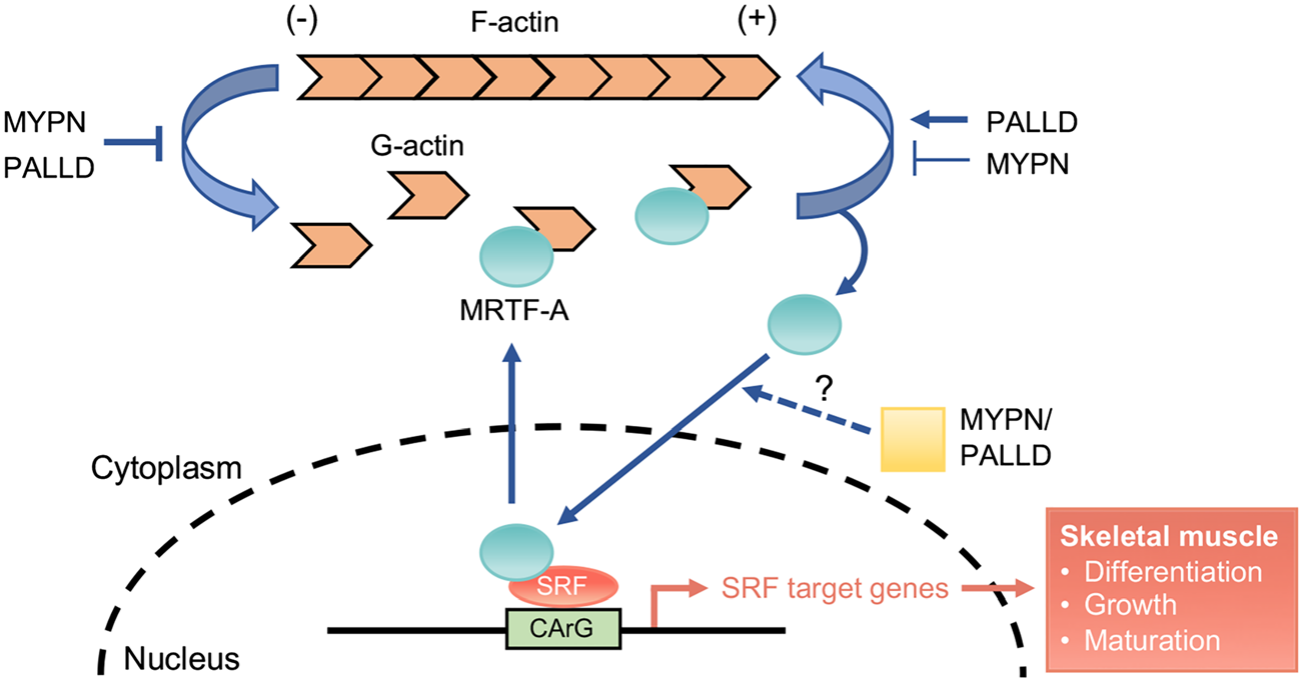
Working model describing how MYPN and PALLD may promote SRF signalling. The SRF coactivator MRTF‐A is sequestered in the cytoplasm through binding to G‐actin and incorporation of G‐actin into F‐actin filaments releases MRTF‐A, allowing it to translocate to the nucleus where it binds and activates the SRF transcription factor.^46^ Thus, the nuclear accumulation of MRTF‐A, and consequently SRF activity, is regulated by the F‐actin/G‐actin ratio. MYPN inhibits actin polymerization but strongly prevents actin depolymerization, with the net result of increasing the F‐actin/G‐actin ratio, stimulating the SRF signalling pathway. PALLD both promotes actin polymerization and inhibits actin depolymerization and thus has an even stronger activating effect on the SRF pathway. Both MYPN and PALLD bind to the central region of MRTF‐A, comprising its SAP and LZ domains, possibly further affecting the localization or activity of MRTF‐A.

Like PALLD,^48^ we found that MYPN binds to MRTF‐A and MRTF‐B and colocalizes with MRTF‐A both in the sarcomere and the nucleus, suggesting that MYPN and PALLD may directly affect the shuttling of MRTFs between the cytoplasm and the nucleus. Both proteins bind to the central region of MRTF‐A, comprising the SAP and LZ domains. The LZ motif allows homodimerization and heterodimerization with myocardin and other MRTFs, while SAP domains have been implicated in chromosomal dynamics, nuclear organization, and apoptosis.^46^ In particular, the MRTF‐A SAP domain has been implicated in both SRF‐dependent and SRF‐independent transcription of genes involved in cell proliferation, migration, and metastasis.^51^ In summary, by stabilizing F‐actin and possibly through its binding to MRTFs, MYPN activates SRF, providing a molecular mechanism for the reduced myofibre CSA in MKO mice (*Figure* 10). The significance of the binding of MYPN and PALLD to MRTFs will be subject to future studies.

RNA‐Seq analysis revealed a number of genes that were highly up‐regulated in MKO EDL muscle, including *Sln*, *Ankrd1*, and *Pak1*. SLN regulates SERCA activity and promotes mitochondrial biogenesis and oxidative metabolism in response to increased energy demand, improving endurance and muscle performance.^52^ SLN has been reported to be up‐regulated in mouse models of Duchenne muscular dystrophy and various myopathies,^53^ suggesting that SLN up‐regulation may be a general response to increased metabolic demand under conditions of compromised muscle function to improve oxidative metabolism. Although CARP/Ankrd1 is a target of SRF, its transcript levels were up‐regulated both in MKO muscle and primary cells, while it was unaltered at the protein level. It is possible that the up‐regulation may be related to its interaction with MYPN and general induction in conditions of stress.^54^ PAK1 expression was increased in MKO muscle both at the RNA and protein level, while the PAK1 phosphorylation level was unchanged. PAK1 plays an important role in the regulation of actin cytoskeletal dynamics^55^, ^56^ and has been shown to promote myoblast differentiation and muscle regeneration as well as counteract muscle atrophy during cancer cachexia.^57^, ^58^ Thus, the up‐regulation of PAK1 is not likely to contribute to the reduction in myofibre CSA in MKO mice and may be a compensatory response to the reduced SRF signalling in MKO mice. The unexpected activation of Akt and the mitogen‐activated protein kinases ERK and JNK in MKO mice, which all promote muscle growth,^59^, ^60^ may likewise be a compensatory response to counteract the reduced myofibre CSA or simply due to delayed growth of MKO mice (except for ERK, they are only up‐regulated in 4‐week‐old MKO mice).

The MKO mouse model is particularly relevant in light of the recent identification of biallelic loss‐of‐function mutations associated with NM,^11^ cap myopathy,^10^ and congenital myopathy with hanging big toe and ultrastructural changes, including Z‐line fragmentation and disruption of the regular square pattern of the Z‐line, possible early manifestations of Z‐line destabilization.^12^ TEM of MKO muscle showed normal sarcomere organization without nemaline rod bodies or caps. However, MKO mice developed progressive Z‐line widening from 8 months of age. Furthermore, after downhill running, we observed Z‐line streaming and regenerating myofibres with centralized nuclei in MKO muscle, while no damage or signs of regeneration were present in WT mice subjected to the same exercise protocol. Thus, MYPN is important for the maintenance of Z‐line integrity during aging and in response to exercise, suggesting that exercise may aggravate the disease in patients carrying biallelic *MYPN* mutations. Increased muscle damage in MKO mice was consistent with decreased exercise capability of MKO mice already from the second day of downhill running. On the other hand, the reduction in isometric force after repetitive eccentric contractions *in vitro* was similar in MKO and WT mice. The increased injury of MKO mice after downhill running may be due to the fact that MKO muscle generate less absolute force compared with WT muscle and thus must be activated to a higher relative level than WT muscle to achieve the same absolute force required for locomotion, thus resulting in higher injury.^61^

The phenotype of the MKO mice is distinct from the phenotype of homozygous knock‐in mice, carrying the MYPN p.Q526X nonsense mutation (MYPN^Q526X^),^62^ equivalent to the human c.1585C > T mutation, which is causative for RCM. While heterozygous MYPN^Q526X^ mice express a truncated 65 kDa MYPN protein and develop RCM, recapitulating the human disease, homozygous MYPN^Q526X^ mice show impaired MYPN transcription and were thus considered a model of MYPN gene ablation. However, in contrast to MKO mice, MYPN^Q526X^ mice did not exhibit reduced myofibre CSA and did not appear to show muscle weakness,^11^ although biomechanical studies have not been performed to verify this. Furthermore, even though MYPN^Q526X^ mice had well‐organized sarcomeres, some Z‐line streaming and the presence of small nemaline‐like bodies reminiscent of mild NM were reported. The discrepancy between the two models suggests that the MYPN^Q526X^ mouse model cannot be considered a knockout model and a small amount of truncated 65 kDa MYPN protein is likely responsible for the observed phenotype, suggesting that truncated MYPN protein rather than MYPN ablation is responsible for the NM phenotype. This is consistent with the detection of 5′ MYPN mRNA transcripts both in heart and skeletal muscle of homozygous MYPN^Q526X^ mice as well as a small amount of truncated 65 kDa MYPN protein in the heart of homozygous MYPN^Q526X^ mice,^62^ suggesting that trace amounts of 65 kDa MYPN protein are likely to be present also in skeletal muscle.

Muscle weakness in NM has been associated with decreased thin filament length, reduced actomyosin interaction, or altered calcium sensitivity,^63^ which is not the case in MKO mice in which reduced myofibre CSA is responsible for the observed muscle weakness. Considering that patients carrying biallelic MYPN mutations have strongly reduced MYPN expression and often show fibre size variability and atrophy, it would be interesting to determine whether the muscle weakness may at least partly be due to reduced myofibre CSA and SRF signalling.

## Author contributions

M.L.B. and M.L. conceived the study. M.L.B., M.L., M.R.B., R.L.L., and P.K.L. designed experiments. M.C.F., D.L.Y., M.C., V.K.K., G.M., A.V., A.G., I.P., R.K., P.K.L., and M.L.B. performed experiments. S.S., M.M., M.C., and R.L. performed bioinformatics analysis. M.C.F., M.C., R.L.L., and M.L.B. analysed data. M.L.B., M.L., M.R.B., R.L.L., P.K.L., and V.N. provided financial support and conceptual advice. M.L.B., M.L., and M.R.B. wrote the manuscript. All authors read, commented, and approved the final manuscript.

## Conflict of interest

None declared.

## Supporting information


**Figure S1.** **Generation of myopalladin knockout mice.** (A) Targeting strategy for generation of MYPN knockout (MKO) mice. A restriction map of the relevant genomic region of *Mypn* is shown on top, the targeting construct is shown in the middle, and the mutated locus after recombination is shown at the bottom. The grey box indicates exon 1. Neo, neomycin resistance gene. (B) Detection of wild‐type (WT) and targeted alleles by Southern blot analysis after digestion with *Mfe*I using the probe shown in (A). Het, heterozygous. (C) Northern blot analysis showing the successful ablation of MYPN in left ventricle (LV) and *tibialis anterior* (TA) muscle from MKO and WT mice. (D) Detection of MYPN protein by Western blot analysis. GAPDH antibody was used as loading control.
**Figure S2. Immunofluorescence and transmission electron microscopy analysis of MKO mice.** (A) Examples of immunofluorescence stainings for myosin heavy chain isoforms (MHC 2A, 2B, and all MHC isoforms except 2X; green) and laminin (red) on cryosectioned *extensor digitorum longus* (EDL) and soleus (MHC 1) muscle. (B) Low magnification transmission electron micrographs from EDL muscle of 8‐month‐old MKO and WT mice showing normal sarcomere organization.
**Figure S3. Mechanical methods.** (A) Sample record of length change (lower trace) during isotonic contraction against a load of 0.5 *T*_0_ (upper trace) from WT EDL muscle. The vertical line indicates the stimulus start. Inset, sample records of length changes during isotonic contraction against different loads as indicated by the values close to the traces. (B) *T*_1_ relations for four pCa values obtained from a single fiber of a WT EDL muscle. The relations were obtained by plotting the extreme force attained at the end of the length step, *T*_1_ (relative to *T*_0_ at pCa = 4.50) *vs*. the length step amplitude. Inset, force response (lower trace) to a length step release of 1 nm (upper trace) at pCa = 4.50 (horizontal line below force response, force baseline).
**Figure S4. Immunofluorescence analysis following BaCl_2_ injection in mouse TA muscle from MKO and WT mice.** Representative laminin (red) and DAPI (blue) stainings of cryosectioned TA muscle from BaCl_2_‐injected muscle *vs*. control (Ctrl) muscle 10 and 21 days after injection of MKO and WT mice.
**Figure S5. qRT‐PCR and western blot analyses on C2C12 cells and primary myoblast cultures derived from MKO and WT mice.** (A) qRT‐PCR analysis for *Mypn* on C2C12 cells during proliferation and at different stages following induction of differentiation. β‐actin was used for normalization (*n* = 3 per group from 3 independent experiments). Data are represented as mean ± SEM. ***P < 0.001 *vs*. day 0 of differentiation; ANOVA. (B) Western blot and densitometric analysis for MYPN on C2C12 cells during proliferation and at different stages following induction of differentiation. The blot is representative of 3 replicates per group. β‐actin was used as loading control. Data are represented as mean ± SEM. ***P < 0.001 *vs.* day 0 of differentiation; one‐way ANOVA. (C) qRT‐PCR on C2C12 cells 2 and 3 days after transfection with MYPN or control vector for quantification of levels of *Mypn* and *Palld* transcripts, encoding the most common PALLD isoforms, as well as myogenic markers (*n* = 3 replicates per group from 3 independent experiments). GAPDH was used for normalization. Data are represented as mean ± SEM. *P < 0.05, **P < 0.01, ***P < 0.001; two‐way ANOVA. (D) Western blot and densitometric analyses for proteins involved in muscle growth and atrophy on cell lysate from proliferating (Prol) and differentiating (Diff) myoblasts derived from MKO and WT mice. The blots are representatives of 3 replicates per group from 3 independent experiments. GAPDH was used as loading control. Data are represented as mean ± SEM. *P < 0.05; **P < 0.01; ***P < 0.001; two‐way ANOVA.
**Figure S6. Western blot analysis on TA muscle from MKO and WT mice.** (A) Western blot analyses on TA muscle lysate from 4‐ and 8‐week‐old MKO and WT littermate control mice for MYPN‐interacting proteins and proteins involved in muscle signaling pathways. α‐Tubulin was used as loading control. The blots are representatives of 3 replicates per group. (B) Densitometric analysis. Data are represented as mean ± SEM. *P < 0.05, **P < 0.01, ***P < 0.001; Student's *t*‐test.
**Figure S7. Immunofluorescence stainings for MYPN‐interacting proteins on TA muscle from 10‐week‐old MKO and WT mice.** DAPI is shown in blue.
**Figure S8. Efficient Ad‐SRF‐VP16 infection of myoblasts.** Fluorescence microscopy picture of primary myoblast cultures 40 hours after infection with Ad‐SRF‐VP16 and induction of differentiation, showing that the cells were efficiently infected. DAPI is shown in blue.
**Table S1. Oligos used for qRT‐PCR and clonings.**
**Table S2. Antibodies used for Western blot analysis and immunostainings.**

**Table S3. List of differentially expressed genes in *tibialis anterior* (TA) muscle from 2‐week‐old myopalladin knockout (MKO) *vs*. wild‐type (WT) mice resulting from RNA‐sequencing analyses.**

**Table S4. List of differentially expressed genes in *tibialis anterior* (TA) muscle from 4‐week‐old myopalladin knockout (MKO) *vs*. wild‐type (WT) mice resulting from RNA‐sequencing analyses.**
