# Supplementary material for: Myopalladin promotes muscle growth through modulation of the serum response factor pathway
Source: J Cachexia Sarcopenia Muscle. 2019 Oct 24;11(1):169–94. doi: 10.1002/jcsm.12486 (PMC7015241; doi:10.1002/jcsm.12486)
Supplement: Supplementary file 1 — Figure S1. Generation of myopalladin knockout mice. (A) Targeting strategy for generation of MYPN knockout (MKO) mice. A restriction map of the relevant genomic region of Mypn is shown on top, the targeting construct is shown in the middle, and the mutated locus after recombination is shown at the bottom. The grey box indicates exon 1. Neo, neomycin resistance gene. (B) Detection of wild‐type (WT) and targeted alleles by Southern blot analysis after digestion with MfeI using the probe shown in (A). Het, heterozygous. (C) Northern blot analysis showing the successful ablation of MYPN in left ventricle (LV) and tibialis anterior (TA) muscle from MKO and WT mice. (D) Detection of MYPN protein by Western blot analysis. GAPDH antibody was used as loading control. Figure S2. Immunofluorescence and transmission electron microscopy analysis of MKO mice. (A) Examples of immunofluorescence stainings for myosin heavy chain isoforms (MHC 2A, 2B, and all MHC isoforms except 2X; green) and laminin (red) on cryosectioned extensor digitorum longus (EDL) and soleus (MHC 1) muscle. (B) Low magnification transmission electron micrographs from EDL muscle of 8‐month‐old MKO and WT mice showing normal sarcomere organization. Figure S3. Mechanical methods. (A) Sample record of length change (lower trace) during isotonic contraction against a load of 0.5 T 0 (upper trace) from WT EDL muscle. The vertical line indicates the stimulus start. Inset, sample records of length changes during isotonic contraction against different loads as indicated by the values close to the traces. (B) T 1 relations for four pCa values obtained from a single fiber of a WT EDL muscle. The relations were obtained by plotting the extreme force attained at the end of the length step, T 1 (relative to T 0 at pCa = 4.50) vs. the length step amplitude. Inset, force response (lower trace) to a length step release of 1 nm (upper trace) at pCa = 4.50 (horizontal line below force response, force baseline). Figure S4. Immun [file JCSM-11-169-s001.pdf]

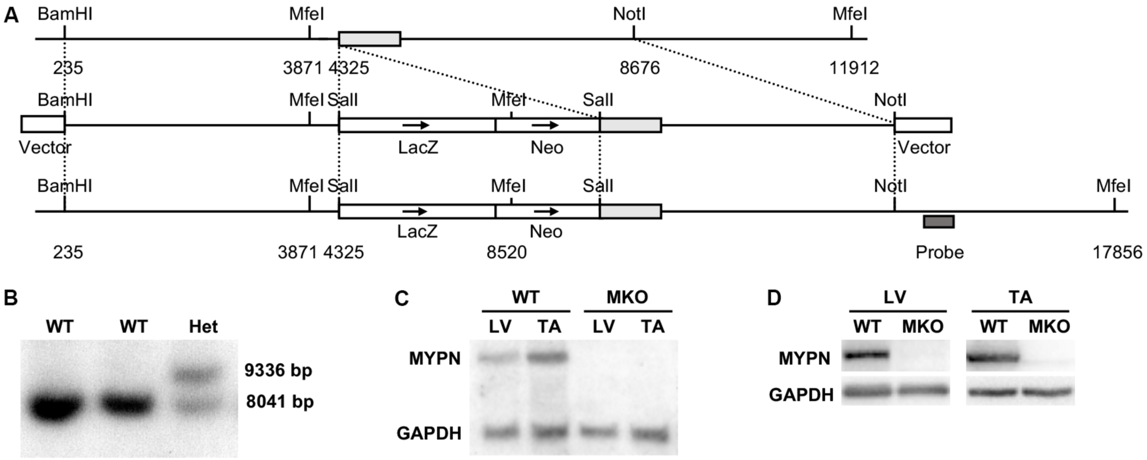

Figure S1. Generation of myopalladin knockout (MKO) mice. (A) Targeting strategy for generation of MKO mice. A restriction map of the relevant genomic region of *Mypn* is shown on top, the targeting construct is shown in the middle, and the mutated locus after recombination is shown at the bottom. The grey box indicates exon 1. Neo, neomycin resistance gene. (B) Detection of wild-type (WT) and targeted alleles by Southern blot analysis after digestion with *Mfe*I using the probe shown in (A). Het, heterozygous. (C) Northern blot analysis showing the successful ablation of MYPN in left ventricle (LV) and *tibialis anterior* (TA) muscle from MKO and WT mice. (D) Detection of MYPN protein by Western blot analysis. GAPDH antibody was used as loading control.

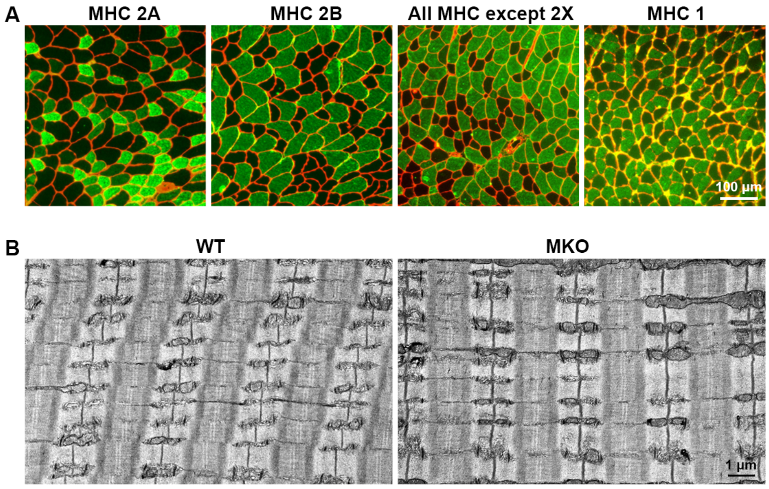

Figure S2. Immunofluorescence and transmission electron microscopy analysis of myopalladin knockout (MKO) mice. (A) Examples of immunofluorescence stainings for myosin heavy chain isoforms (MHC 2A, 2B, and all MHC isoforms except 2X; green) and laminin (red) on cryosectioned *extensor digitorum longus* (EDL) and soleus (MHC 1) muscle. (B) Low magnification transmission electron micrographs from EDL muscle of 8-month-old MKO and wild-type (WT) mice showing normal sarcomere organization.

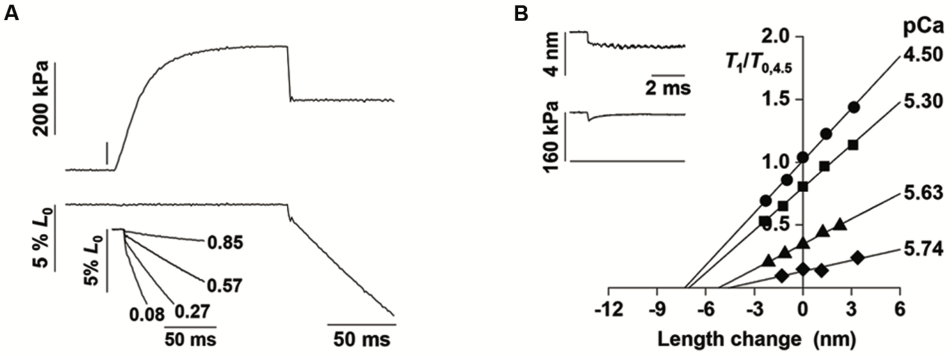

Figure S3. Mechanical methods. (A) Sample record of length change (lower trace) during isotonic contraction against a load of 0.5 T_0 (upper trace) from a wild-type (WT) *extensor digitorum longus* (EDL) muscle. The vertical line indicates the stimulus start. Inset, sample records of length changes during isotonic contraction against different loads as indicated by the values close to the traces. (B) T_1 relations for four pCa values obtained from a single fibre of a WT EDL muscle. The relations were obtained by plotting the extreme force attained at the end of the length step, T_1 (relative to T_0 at pCa = 4.50) *vs*. the length step amplitude. Inset, force response (lower trace) to a length step release of 1 nm (upper trace) at pCa = 4.50 (horizontal line below force response, force baseline).

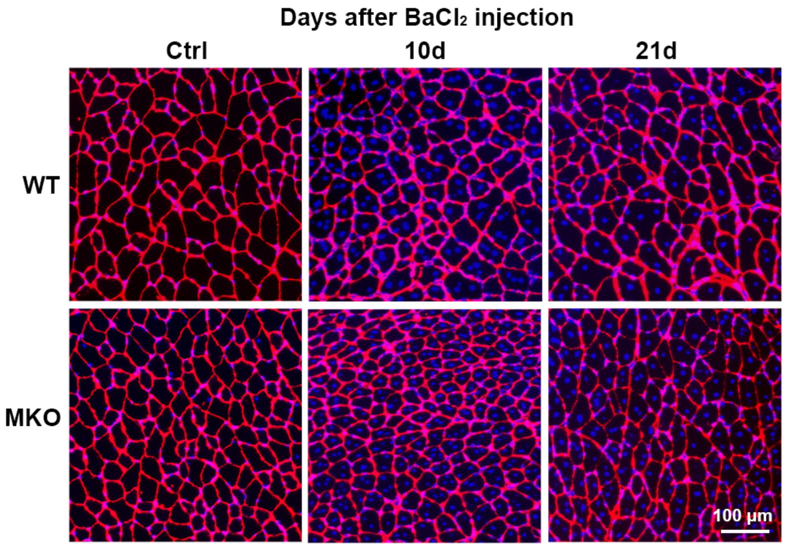

Figure S4. Immunofluorescence analysis following $BaCl_2$ injection in mouse *tibialis anterior* (TA) muscle from myopalladin knockout (MKO) and wild-type (WT) mice. Representative laminin (red) and 4', 6-diamidino-2-phenylindole (DAPI) (blue) stainings of cryosectioned TA muscle from $BaCl_2$-injected muscle vs. control (Ctrl) muscle 10 and 21 days after injection of MKO and WT mice.

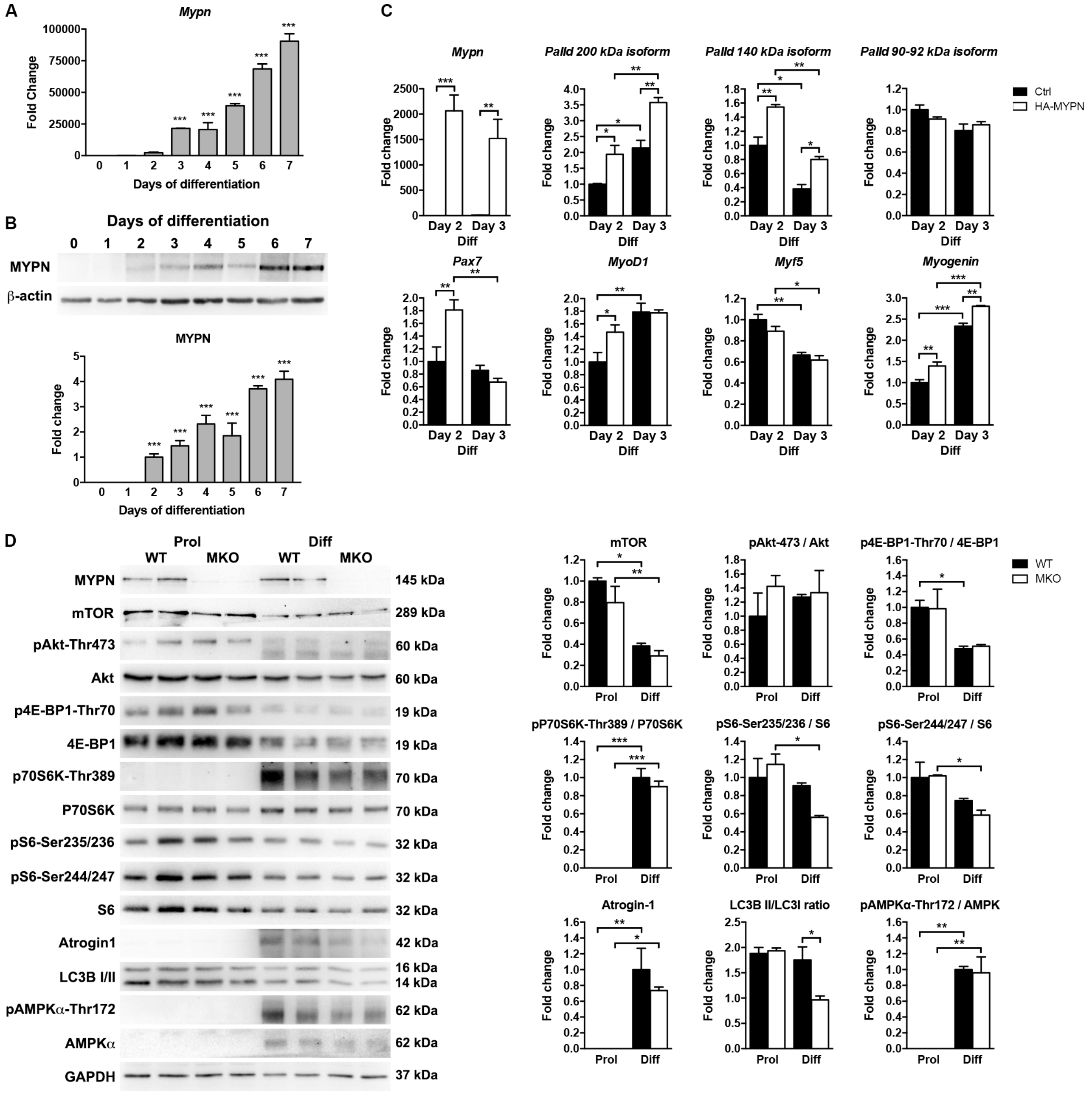

Figure S5. Quantitative real-time PCR (qRT-PCR) and western blot analyses on C2C12 cells and primary myoblast cultures derived from myopalladin knockout (MKO) and wild-type (WT) mice. (A) qRT-PCR analysis for *Mypn* on C2C12 cells during proliferation and at different stages following induction of differentiation. β-actin was used for normalization ($n = 3$ per group from 3 independent experiments). Data are represented as mean ± standard error of the mean (SEM). ***$P < 0.001$ *vs*. day 0 of differentiation; one-way analysis of variance (ANOVA). (B) Western blot and densitometric analysis for myopalladin (MYPN) on C2C12 cells during proliferation and at different stages following induction of differentiation. The blot is representative of 3 replicates per group. β-actin was used as loading control. Data are represented as mean ± SEM. ***$P < 0.001$ vs. day 0 of differentiation; one-way ANOVA. (C) qRT-PCR on C2C12 cells 2 and 3 days after transfection with MYPN or control vector for quantification of levels of *Mypn* and *Palld* transcripts, encoding the most common palladin (PALLD) isoforms, as well as myogenic markers ($n = 3$ replicates per group from 3 independent experiments). GAPDH was used for normalization. Data are represented as mean ± SEM. *$P < 0.05$, **$P < 0.01$, ***$P < 0.001$; two-way ANOVA. (D) Western blot and densitometric analyses for proteins involved in muscle growth and atrophy on cell lysate from proliferating (Prol) and differentiating (Diff) myoblasts derived from MKO and WT mice. The blots are representatives of 3 replicates per group from 3 independent experiments. GAPDH was used as loading control. Data are represented as mean ± SEM. *$P < 0.05$; **$P < 0.01$; ***$P < 0.001$; two-way ANOVA.

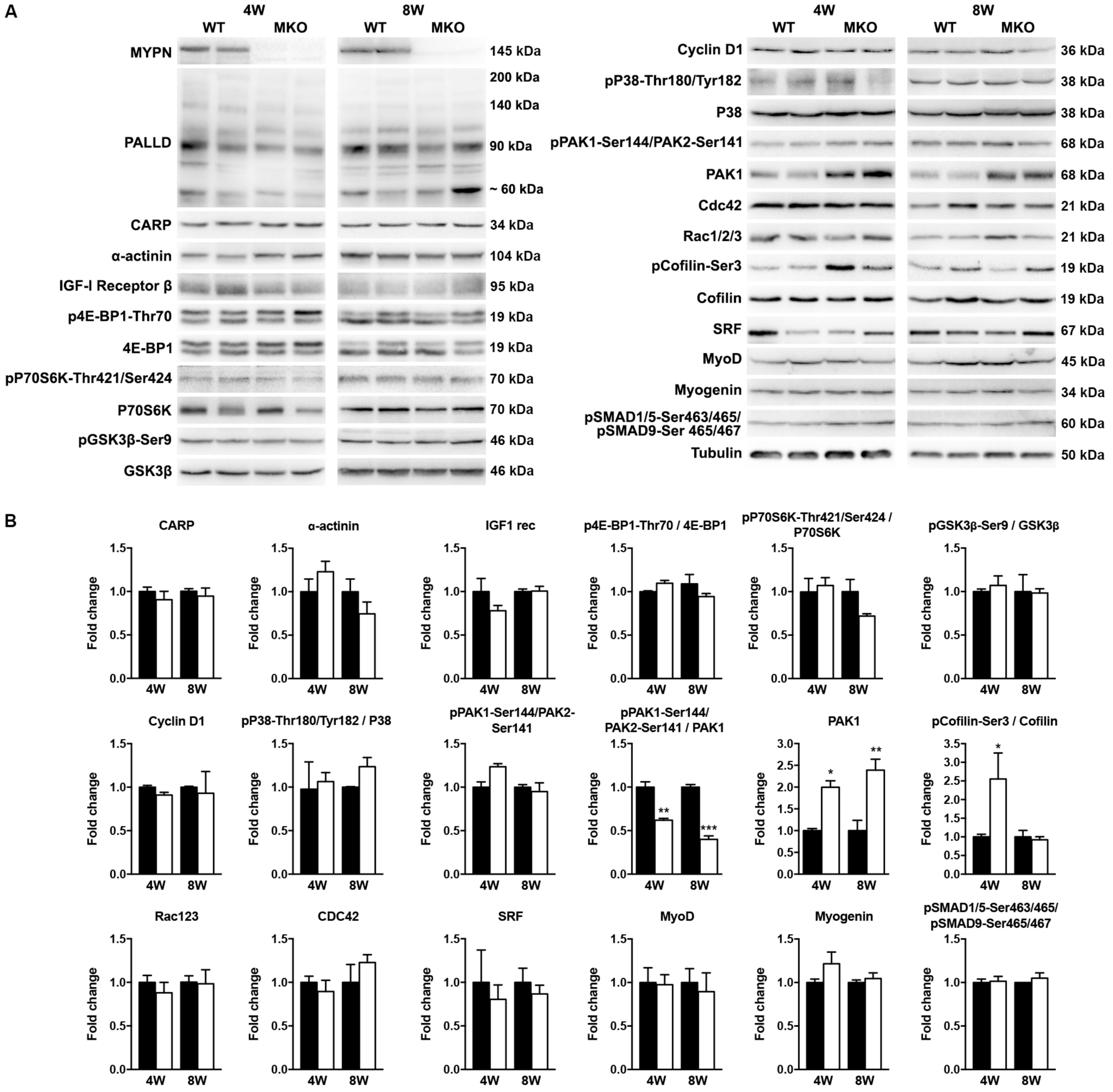

Figure S6. Western blot analysis on *tibialis anterior* (TA) muscle from myopalladin knockout (MKO) and wild-type (WT) mice. (A) Western blot analyses on TA muscle lysate from 4- and 8-week-old MKO and WT littermate control mice for myopalladin (MYPN)-interacting proteins and proteins involved in muscle signaling pathways. α-Tubulin was used as loading control. The blots are representatives of 3 replicates per group. (B) Densitometric analysis. Data are represented as mean ± standard error of the mean. *$P < 0.05$, **$P < 0.01$, ***$P < 0.001$; Student's *t*-test.

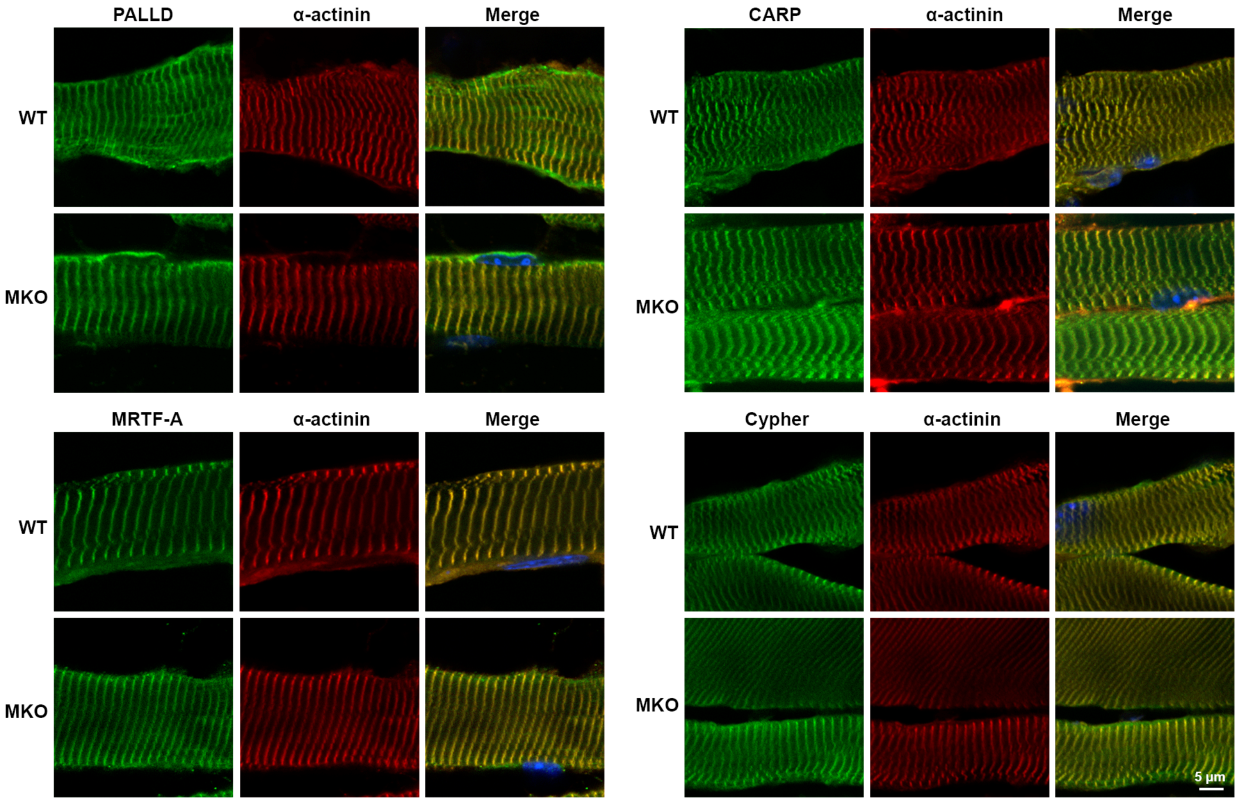

Figure S7. Immunofluorescence stainings for myopalladin (MYPN)-interacting proteins on *tibialis anterior* (TA) muscle from 10-week-old myopalladin knockout (MKO) and wild-type (WT) mice. 4’, 6-diamidino-2-phenylindole (DAPI) is shown in blue.

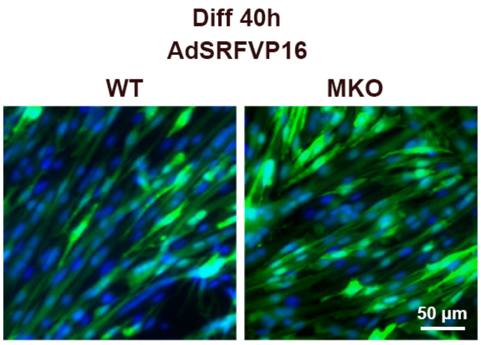

Figure S8. Efficient Ad-SRF-VP16 infection of myoblasts. Fluorescence microscopy picture of primary myoblast cultures 40 hours after infection with Ad-SRF-VP16 and induction of differentiation, showing that the cells were efficiently infected. 4’, 6-diamidino-2-phenylindole (DAPI) is shown in blue.

Table S1. Oligos used for quantitative real-time PCR (qRT-PCR) and clonings.

Primers	Sense	Reverse
**qRT-PCR**		
Mypn	CATGCTTTGCTTCCAACATT	GGCTTCTGGATTCGATTCAT
Palld 200 kDa	CATCCAGAAACTGAGGAGCC	AGCTTTCGCTGTCAGAGTCC
Palld 140 kDa	TGCTGCCTGTGCATTTTCCC	AGCTTTCGCTGTCAGAGTCC
Palld 90 kDa	AGGAGCCCTCGACACCCA	TCCTGTTCCAGGCGCACTTGG
Ankrd1	GCTGGAGCCCAGATTGAA	CTCCACGACATGCCCAGT
Ankrd2	CGTGAGACTCAACCGCTACA	GCAGGCAGCTCATAGTAGGG
Pax7	GGCACAGAGGACCAAGCTC	GCACGCCGGTTACTGAAC
Myf5	CTGCTCTGAGCCCACCAG	GACAGGGCTGTTACATTCAGG
Myod1	AGCACTACAGTGGCGACTCA	GGCCGCTGTAATCCATCAT
Myog	GAGATCCTGCGCAGCGCCAT	CCCCGCCTCTGTAGCGGAGA
Sln	CTGAGGTCCTTGGTAGCCTG	AAGCTAAGGCTCACTGGCTG
Pak1	TGTCTGAGACCCCAGCAGTA	CCCGAGTTGGAGTAACAGGA
Srf	GCTTCACCAGATGGCTGTGATA	AATAAGTGGTGCCGTCCCTTG
Mrtfa	ATGACATGAAGGTGGCAGAG	TGACTTGGTCTTGGTAGGCA
Acta1	AATGAGCGTTTCCGTTGC	ATCCCCGCAGACTCCATAC
Acta2	CTGACAGAGGCACCACTGAA	CATCTCCAGAGTCCAGCACA
Actc1	TGCCGATCGTATGCAAAAGG	GGCCTGCCTCATCATACTCT
Ctgf	TGACCTGGAGGAAAACATTAAGA	AGCCCTGTATGTCTTCACACTG
Cacna2d4	CAGGTGAGGTCTCCAAGAGC	TGTGTTCACTTGGGCATTGT
Myl9	GATAAGGAGGACCTGCACGA	GGAACATGGTGAAGTTGATGG
Myh4	ACAGACTAAAGTGAAAGCC	CTCTCAACAGAAAGATGGAT
Gapdh	TGGCAAAGTGGAGATTGTTGCC	AAGATGGTGATGGGCTTCCCG
**Clonings**		
Pxj-40-mouse PALLD isoform 4 (res. 1-680; BC127081)	atccaagcttctcgag/ATGAGCGCTCTGGCCTCCCG	agatctggtaccctgcagcc/TCACAGGTCTTCACTTTCTACCAAGCC
Pxj40-HA-mouse MYPN FL (res. 1-1315; NM_182992)	atccaagcttctcgag/ATGCAAGAAGACAGCATAGAGGCATCCA	agatctggtaccctgcagcc/TTAAAGTTCATCGCTCTCCACTACACTCC
Pxj40-HA-mouse MYPN C-term (res. 938-1315; NM_182992)	atccaagcttctcgag/TGCATCGCGCCCATCTTTGACAAAAGGCTCAAG	agatctggtaccctgcagcc/TTAAAGTTCATCGCTCTCCACTACACTCC
Pxj40-HA-mouse MYPN Ig3-4 (res. 938-1165; NM_182992)	atccaagcttctcgag/TGCATCGCGCCCATCTTTGACAAAAGGCTCAAG	agatctggtaccctgcagcc/CTTCTTTACCTCTTTGGCTACCACAGTGAGCTCC
Pxj40-HA-mouse MYPN Ig3 (res. 938-1040; NM_182992)	atccaagcttctcgag/TGCATCGCGCCCATCTTTGACAAAAGGCTCAAG	agatctggtaccctgcagcc/TCGGCTTCGAATGGGCAAACCCTGTACCATC
PmCherry-N1-MYPN FL (res. 1-1315; NM_182992)	ctcaagcttcgaattc/ATGCAAGAAGACAGCATAGAGGCATCCA	ggcgaccggtggatcccg/AAGTTCATCGCTCTCCACTACACTCC
pTBMalE3-mouse MYPN Ig3-4 (res. 938-1165; NM_182992)	tacttccaatccaatgca/TGCATCGCGCCCATCTTTGACAAAAG	ttatccacttccaatg/TTAACTCTTCTTTACCTCTTTGGCTACCACAGTGAG
pTBSG-mouse MYPN Ig3 (res. 940-1044; NM_182992)	tacttccaatccaatgcg/GCTCCCATCTTTGACAAGAGACTCAAGC	ttatccacttccaatg/TTAAGCAGAGGTTAGCCGACTGCGAAT
pTBMalE3-mouse MYPN Ig4 (res. 1065-1165; NM_182992)	tacttccaatccaatga/TTTTTCCGACCACATTTCCTGCAG	ttatccacttccaatg/TTAACTCTTCTTTACCTCTTTGGCTACCACAGTGAG
pGBKT7 human MYPN FL (res. 1-1320; NM_182992)	tttcatatg/ATGCAAGACGACAGCATAGAGCTTCTACT	tttggatcc/TTAAAGTTCATCACTCTCCACTACACTCCG
pGBKT7 human MYPN C-term (res. 938-1320; NM_182992)	tttcatatg/CCCACGGGCAAGTGTATTGCTCCCATCTTT	tttggatcc/TTAAAGTTCATCACTCTCCACTACACTCCG
pGBKT7 human MYPN Ig3-4 (res. 944-1155; NM_182992)	catggaggccgaattc/GCTCCCATCTTTGACAAGAGACTCAAGC	gcaggtcgacggatcctca/ATTCTGCCCGGTTTTGTTGGTAGCGATG
pGBKT7-human MYPN Ig3 (res. 944-1048; NM_182992)	catggaggccgaattc/GCTCCCATCTTTGACAAGAGACTCAAGC	gcaggtcgacggatcctca/AGCAGAGGTTAGCCGACTGCGAATG
pGADT7-human MRTF-A/MKL1 FL res. 1-931 (NM_020831)	ggaggccagtgaattc/ATGCCGCCTTTGAAAAGTCCAGC	cgagctcgatggatcc/CTACAAGCAGGAATCCCAGTGCAG
pGADT7-human MRTF-A/MKL1 res. 1-169 (NM_020831)	ggaggccagtgaattc/ATGCCGCCTTTGAAAAGTCCAGC	cgagctcgatggatccccta/ATCCCGGCCCATCGGAAGTTGAG
pGADT7-human MRTF-A/MKL1 res. 170-931 (NM_020831)	ggaggccagtgaattc/TCCAGAGAAATGCTTTTCCTGGCAGAGC	cgagctcgatggatcc/CTACAAGCAGGAATCCCAGTGCAG
pGADT7-human MRTF-A/MKL1 res. 170-287 (NM_020831)	ggaggccagtgaattc/TCCAGAGAAATGCTTTTCCTGGCAGAGC	cgagctcgatggatcccta/GGCCTGGTAGTTGTGGTGCTGC
pGADT7-human MRTF-A/MKL1 res. 608-931 (NM_020831)	ggaggccagtgaattc/CACATAGACCCTTGTGCTGTGGCC	cgagctcgatggatcc/CTACAAGCAGGAATCCCAGTGCAG
pGADT7-human MRTF-A/MKL1 res. 288-611 (NM_020831)	ggaggccagtgaattc/ATCCTGCCTGCCCCGCCAAAG	cgagctcgatggatcccta/AGGGTCTATGTGGTTGGTGGCTGG
pGADT7-human MRTF-A/MKL1 res. 288-514 (NM_020831)	ggaggccagtgaattc/ATCCTGCCTGCCCCGCCAAAG	cgagctcgatggatcccta/CCCCCCAGGGCTCAGGCAAC
pGADT7-human MRTF-A/MKL1 res. 347-611 (NM_020831)	ggaggccagtgaattc/CTGGACGACATGAAGGTGGCAGAG	Cgagctcgatggatcccta/AGGGTCTATGTGGTTGGTGGCTGG
pGADT7-human MRTF-A/MKL1 res. 382-611 (NM_020831)	ggaggccagtgaattc/GACCAAATCAGCCCTGTGCCAGG	cgagctcgatggatcccta/AGGGTCTATGTGGTTGGTGGCTGG
pGADT7-human MRTF-A/MKL1 res. 347-514 (NM_020831)	ggaggccagtgaattc/CTGGACGACATGAAGGTGGCAGAG	cgagctcgatggatcccta/CCCCCCAGGGCTCAGGCAAC

Vector specific sequence is written in lowercase letters. FL, full-length

Table S2. Antibodies used for western blot analysis and immunostainings.

Antibody	Source	Code	WB conc.	IF conc.
Rabbit polyclonal anti-MYPN	Yamamoto et al., 2013a[1]	N/A	1:1000	
Rabbit polyclonal anti-PALLD 621	Pogue-Geile, 2006[2]	N/A	1:500	1:30
Rabbit polyclonal anti-CARP/Ankrd1	Proteintech	Cat# 11427-AP	1:1000	1:20
Mouse monoclonal anti-α-actinin (Clone EA-53)	Sigma-Aldrich	Cat# A7811	1:50.000	1:250
Rabbit polyclonal anti-Cypher	Zhou et al., 2013[3]	N/A		1:50
Rabbit polyclonal anti-desmin	Abcam	Cat# Ab8592		1:80
Rabbit polyclonal anti-MRTF-A/MKL1	Sigma-Aldrich	Cat# AV37504		1:30
Mouse monoclonal anti-ubiquitinated proteins (clone FK2)	Merck Millipore	Cat# 04-263	1:1000	
Mouse monoclonal anti-puromycin (clone 12D10)	Merck Millipore	Cat# MABE343	1: 25.000	
Rabbit polyclonal anti-IGF-I Receptor β	Cell Signaling Technology	Cat# 3027	1:500	
Rabbit polyclonal anti-AKT-Thr308	Cell Signaling Technology	Cat# 2965	1:500	
Rabbit polyclonal anti-AKT-Ser473	Cell Signaling Technology	Cat# 4060	1:500	
Rabbit polyclonal anti-AKT	Cell Signaling Technology	Cat# 9272	1:1000	
Rabbit polyclonal anti-p4E-BP1-Thr70	Cell Signaling Technology	Cat# 9455	1:500	
Rabbit polyclonal anti-4E-BP1	Cell Signaling Technology	Cat# 9644	1:500	
Rabbit polyclonal anti-pP70-S6K-Thr389	Cell Signaling Technology	Cat# 9206	1:500	
Rabbit polyclonal anti-pP70-S6K-Thr421/Ser424	Cell Signaling Technology	Cat# 9204	1:500	
Rabbit polyclonal anti-P70-S6K	Cell Signaling Technology	Cat# 2708	1:500	
Rabbit polyclonal anti-pS6-Ser235/236	Cell Signaling Technology	Cat# 2211	1:500	
Rabbit polyclonal anti-pS6-Ser244/247	Thermo Fisher Scientific	Cat# 44-923G	1:500	
Rabbit polyclonal anti-S6	Cell Signaling Technology	Cat# 2317	1:500	
Rabbit polyclonal anti-GSK3β-S9	Cell Signaling Technology	Cat# 5558	1:500	
Rabbit polyclonal anti-GSK3β	Cell Signaling Technology	Cat# 9315	1:1000	
Rabbit polyclonal anti-Cyclin D1	Cell Signaling Technology	Cat# 2922	1:500	
Rabbit polyclonal anti-pP38-Thr180/Tyr182	Cell Signaling Technology	Cat# 4631	1:500	
Rabbit polyclonal anti-P38	Santa Cruz Biotechnology	Cat# 7972	1:500	
Rabbit polyclonal anti-pErk1/2-Thr202/Tyr204	Cell Signaling Technology	Cat# 4370	1:500	
Rabbit polyclonal anti-Erk1/2	Santa Cruz Biotechnology	Cat# sc-514302	1:1000	
Rabbit polyclonal anti-pJNK-Thr183/Tyr185 (G-7)	Santa Cruz Biotechnology	Cat# sc-6254	1:500	
Rabbit polyclonal anti-JNK (D-2)	Santa Cruz Biotechnology	Cat# sc-7345	1:500	
Rabbit polyclonal anti-pPAK1-Ser144/PAK2-Ser141	Cell Signaling Technology	Cat# 2606	1:500	
Rabbit polyclonal anti-PAK1	Cell Signaling Technology	Cat# 2602	1:1000	
Rabbit polyclonal anti-Cdc42	Cell Signaling Technology	Cat# 2466	1:500	
Rabbit polyclonal anti-Rac1/2/3	Cell Signaling Technology	Cat# 2465	1:500	
Rabbit polyclonal anti-pCofilin-Ser3	Cell Signaling Technology	Cat# 3313	1:500	
Rabbit polyclonal anti-Cofilin	Cell Signaling Technology	Cat# 3318	1:500	
Rabbit monoclonal anti-SRF (G-20)	Santa Cruz Biotechnology	Cat# sc-335	1:1000	
Rabbit polyclonal anti-pSMAD1/5-Ser463/465/9-Ser465/467	Cell Signaling Technology	Cat# 9511	1:500	
Rabbit polyclonal anti-MAFbx/Atrogin 1	Santa Cruz Biotechnology	Cat# sc-27644	1:500	
Rabbit polyclonal anti-LC3B	Sigma-Aldrich	Cat# L7543	1:500	
Rabbit polyclonal anti-pAMPKα-Thr172	Cell Signaling Technology	Cat# 2535	1:500	
Rabbit polyclonal anti-AMPKα	Cell Signaling Technology	Cat# 2532	1:500	
Rabbit polyclonal anti-PAX7	Developmental studies hybridoma bank	Cat# PAX7	1:500	
Rabbit polyclonal anti-Myf5 (C-20)	Santa Cruz Biotechnology	Cat# sc-302	1:500	
Rabbit polyclonal anti-MyoD (M-318)	Santa Cruz Biotechnology	Cat# sc-760	1:500	
Mouse monoclonal anti-Myogenin	Developmental studies hybridoma bank	Cat# F5D	1:500	
Rabbit polyclonal anti-laminin	Sigma-Aldrich	Cat# L9393		1:50
Mouse monoclonal anti-Myosin heavy chain, all but 2x	Developmental studies hybridoma bank	Cat# BF-35		1:10
Mouse monoclonal anti-Myosin heavy chain 2A	Developmental studies hybridoma bank	Cat# SC-71		1:1
Mouse monoclonal anti-Myosin heavy chain 2B	Developmental studies hybridoma bank	Cat# BF-F3		1:10
Mouse monoclonal anti-Myosin heavy chain (Slow)	Leica Biosystems (Novocastra)	Cat# NCL-MHCs		1:20
Rabbit polyclonal anti-HA	Sigma-Aldrich	Cat# H6908	1: 1000	
Mouse monoclonal anti-FLAG (clone M2)	Sigma-Aldrich	Cat# F3165	1: 20.000	
Rabbit monoclonal anti-α-tubulin	Abcam	Cat# Ab176560	1:4000	
Goat polyclonal anti-α-actin	Santa Cruz Biotechnology	Cat# sc-1615	1:2000	
Rabbit polyclonal anti-GAPDH	Proteintech	Cat# 10494-1-AP	1: 15.000	
Goat anti-mouse IgG (H + L) Highly-cross Adsorbed Secondary antibody, Alexa Fluor 488-conjugated IgG	Thermo Fisher Scientific	Cat# A11029	1:500	
Goat anti-rabbit IgG (H + L) Highly-cross Adsorbed Secondary antibody, Alexa Fluor 488	Thermo Fisher Scientific	Cat# A11034	1:500	
Goat anti-mouse IgG (H + L) Highly-cross Adsorbed Secondary antibody, Alexa Fluor 568	Thermo Fisher Scientific	Cat# A11031	1:500	
Goat anti-rabbit IgG (H + L) Highly-cross Adsorbed Secondary antibody, Alexa Fluor 568	Thermo Fisher Scientific	Cat# A11036	1:500	

Goat anti-mouse IgG (H + L) Highly-cross Adsorbed Secondary antibody, Alexa Fluor 647	Thermo Fisher Scientific	Cat# A21236	1:500	
Goat anti-rabbit IgG (H + L) Highly-cross Adsorbed Secondary antibody, Alexa Fluor 647	Thermo Fisher Scientific	Cat# A21245	1:500	
Goat anti-rabbit IgG Horseradish Peroxidase (HRP)	Thermo Fisher Scientific	Cat# 31460	1:500	
Goat anti-mouse IgG-HRP	Thermo Fisher Scientific	Cat# 31430	1:500	
Donkey anti-goat IgG-HRP	Santa Cruz Biotechnology	Cat# sc-2020	1:2000	

WB, western blot analysis; IF, immunofluorescence staining

References

1. Yamamoto DL, Vitiello C, Zhang J, Gokhin DS, Castaldi A, Coulis G, et al. The nebulin SH3 domain is dispensable for normal skeletal muscle structure but is required for effective active load bearing in mouse. *J Cell Sci* 2013;**126**:5477-5489.
2. Pogue-Geile KL, Chen R, Bronner MP, Crnogorac-Jurcevic T, Moyes KW, Dowen S, et al. Palladin mutation causes familial pancreatic cancer and suggests a new cancer mechanism. *PLoS Med* 2006;**3**:e516.
3. Zhou Q, Chu PH, Huang C, Cheng CF, Martone ME, Knoll G, et al. Ablation of Cypher, a PDZ-LIM domain Z-line protein, causes a severe form of congenital myopathy. *J Cell Biol* 2001;**155**:605-612.
